# A dynamic combinatorial library for biomimetic recognition of dipeptides in water

**DOI:** 10.3762/bjoc.16.131

**Published:** 2020-07-02

**Authors:** Florian Klepel, Bart Jan Ravoo

**Affiliations:** 1 Organic Chemistry Institute and Center for Soft Nanoscience, Westfälische Wilhelms-Universität Münster, Correnstraße 40, 48149 Münster, Germany

**Keywords:** artificial receptor, dynamic combinatorial chemistry, dynamic covalent chemistry, molecular recognition, thiolate–disulfide exchange

## Abstract

Small peptides are involved in countless biological processes. Hence selective binding motifs for peptides can be powerful tools for labeling or inhibition. Finding those binding motifs, especially in water which competes for intermolecular H-bonds, poses an enormous challenge. A dynamic combinatorial library can be a powerful method to overcome this issue. We previously reported artificial receptors emerging form a dynamic combinatorial library of peptide building blocks. In this study we aimed to broaden this scope towards recognition of small peptides. Employing CXC peptide building blocks, we found that cyclic dimers of oxidized **CFC** bind to the aromatic peptides **FF** and **YY** (*K* ≈ 229–702 M^−1^), while **AA** binds significantly weaker (*K* ≈ 65–71 M^−1^).

## Introduction

Peptides are one of the most abundant and structurally versatile motifs in nature. Thus, selective binders for peptides are extremely desirable but also highly challenging to design. Early developments in artificial peptide recognition go back to the 1970’s when it was discovered that crown ethers can bind to ammonium functions, such as protonated amines in peptides [1]. Further such binding motifs were developed and if arranged correctly can be used to synthesize artificial receptors with high affinity [2–3]. Schmuck et al. have been hugely successful in designing artificial peptide receptors [4–5]. For example, they combined a carboxylate binding site with an aromatic bowl-shaped cavity, just the right size for a methyl group [6]. This receptor was able to bind Ac-AA (*K*_2_ = 3.1 × 10^4^ M^−1^) with a roughly 10-fold higher affinity than Ac-GG. However, constructing binders in this fashion requires a design on a case by case basis, as well as a high synthetical effort for each new iteration. A more general approach for the development of artificial receptors can be the use of a dynamic combinatorial library (DCL). In a DCL a large variety of molecules is generated by a dynamic exchange of building blocks. The dynamic nature of those exchange reactions is essential since it allows the library to be under thermodynamic control. Therefore, the equilibrium responds to stimuli such as addition of a template, which would stabilize a suitable receptor and thus amplifies its formation. This strategy has led to the discovery of many artificial receptors [7–10], with thiolate disulfide exchange being one of the most prominent reactions [11–14]. Notably, while (pseudo)peptides are a common motif for DCL building blocks [7,11,15–16] very few DCLs use peptide templates. Still et al. showed early on that peptide receptors emerging from DCLs are possible. They screened for a suitable peptide (Ac-(ʟ)-Pro-(ʟ)-Val-(ᴅ)-Val) for a pre-existing receptor (*K* ≈ 10^4^–10^5^ M^−1^) first and proved that it can emerge from a DCL afterwards. Despite these encouraging results no further research was done [17]. Additionally, DCLs as a whole can be used to sense molecules. Buryak and Severin developed a DCL composed of Cu^2+^, Ni^2+^ complexes. The addition of dipeptides caused characteristic spectral changes by ligand-exchange reactions [18].

We previously used a peptide derived DCL for the development of artificial carbohydrate receptors [19–20]. An intriguing example derived from that work is the parallel dimer of **CHC** (**p(CHC)_2_**), which binds two molecules of the neurotransmitter *N*-acetylneuraminic acid (NANA) in a cooperative fashion (*K*_1_ = 143, *K*_2_ = 5.1 × 10^3^ M^−1^). Recently we rationalized that since our peptide building blocks consist of the same binding motifs as binding proteins (amines, carboxyl groups, amides, amino acid side chains), our DCL should be applicable to a similar scope. In order to show that our system offers a general approach to find binders for biomolecules in water we choose to test our DCL against peptide templates.

## Results and Discussion

**Scheme 1:**
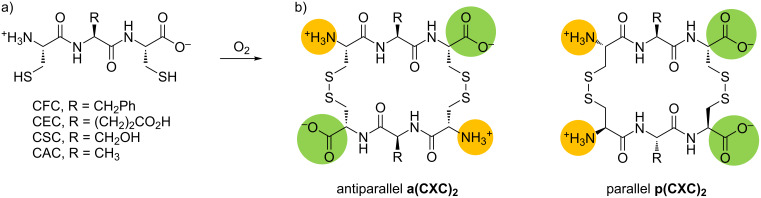
a) Building blocks included in this study. b) Antiparallel and parallel constitutional isomers of tripeptide dimers are formed after oxidation.

We started from our established CXC peptide building block design (single-letter code) were the terminal amino acids are cysteine (C) and the X can be any amino acid (Scheme 1a). This allows for rapid synthesis of various new building bocks by standard Fmoc based SPPS using Trt protecting groups for cysteine, which are subsequently cleaved (see Supporting Information File 1 for details). It also enables easy incorporation of nonnatural amino acids if desired [21].

**Figure 1:**
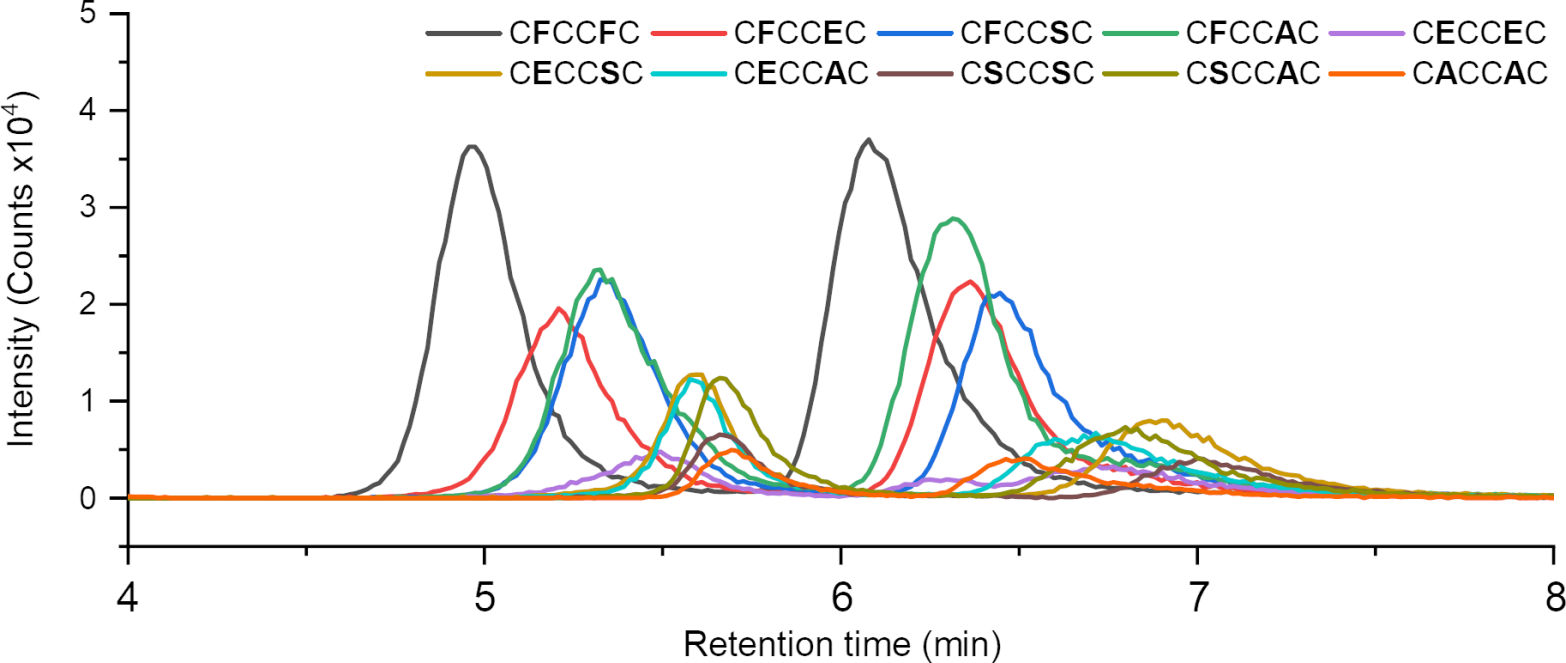
HPLC–MS chromatograms of a reference library for all possible tripeptide dimers ([M + H]^+^ ions).

We prepared a DCL by dissolving **CFC**, **CEC**, **CSC** and **CAC** (C for cysteine, F for phenylalanine, E for glutamic acid, S for serine, A for alanine) in an equimolar ratio in a slightly alkaline buffer (pH 7.4). An excess of our target molecules, the simple dipeptides **AA**, **FF** and **YY** (Y for tyrosine), were added to one sample each. An additional sample without dipeptide addition served as reference. After several hours of stirring in an open vial the thiol functions of the cysteines are converted quantitively to disulfides by oxidation with oxygen. Until fully oxidized library members can exchange building blocks in a thiol–disulfide exchange reaction. While a closed monomer can be observed in the beginning (Figure S1, Supporting Information File 1), exclusively cyclic tripeptide dimers can be found after equilibration. We note that this simple mixture of 4 peptides contains 10 different homo- and heterodimer combinations. In addition, since our building blocks are asymmetrical, parallel and antiparallel constitutional isomers are formed (Scheme 1b), which raises the total amount of different receptor candidates to 20. The exchange was subsequently quenched by protonation of the thiolates and the samples were analyzed by HPLC–MS . Pre-separation was performed on a ZIC-HILIC column, which is known for good peptide selectivity [22]. However, complete separation of all compounds could not be achieved due to the high complexity of the library and the structural similarity of its members. Hence ESI-TOF mass spectrometry data can be interpreted qualitatively but not quantitatively. A full reference sample is shown in Figure 1. Each chromatogram represents a single combination of cyclic tripeptide dimer. Each peptide dimer has two peaks because of the two constitutional isomers. Note that intensities between different compounds are not directly comparable in mass spectrometry.

**Figure 2:**
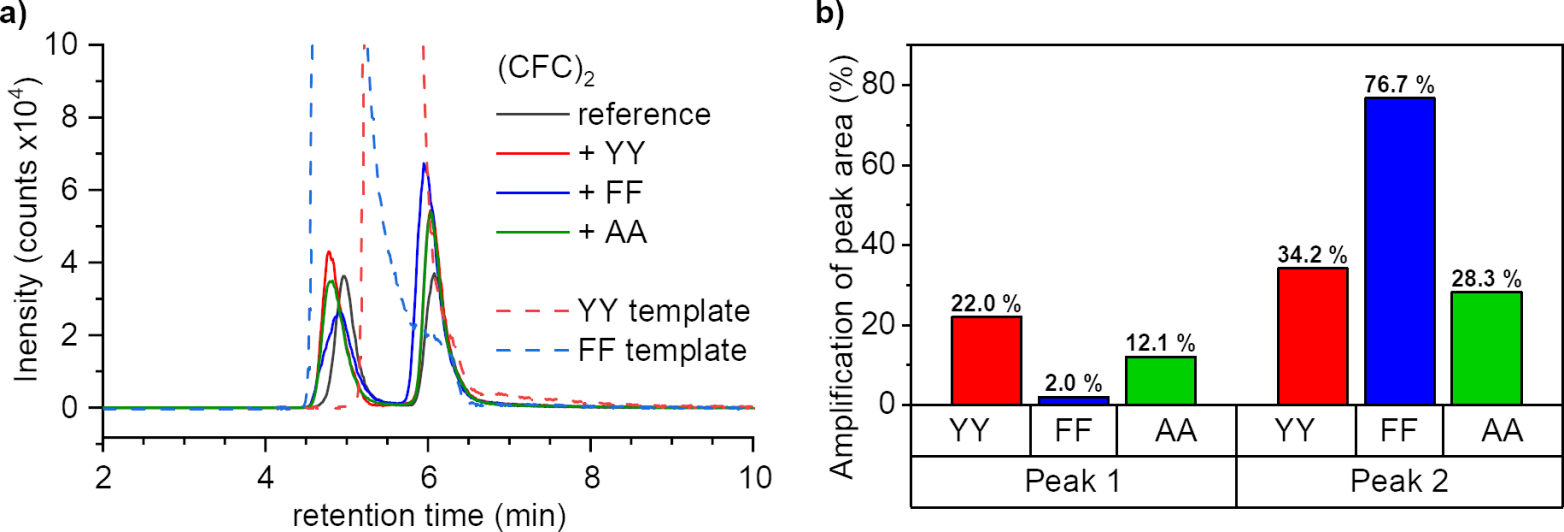
a) HPLC–MS chromatograms of the dimers **(CFC)_2_** and templates **YY** and **FF**. b) Amplification of the peak areas.

**Scheme 2:**
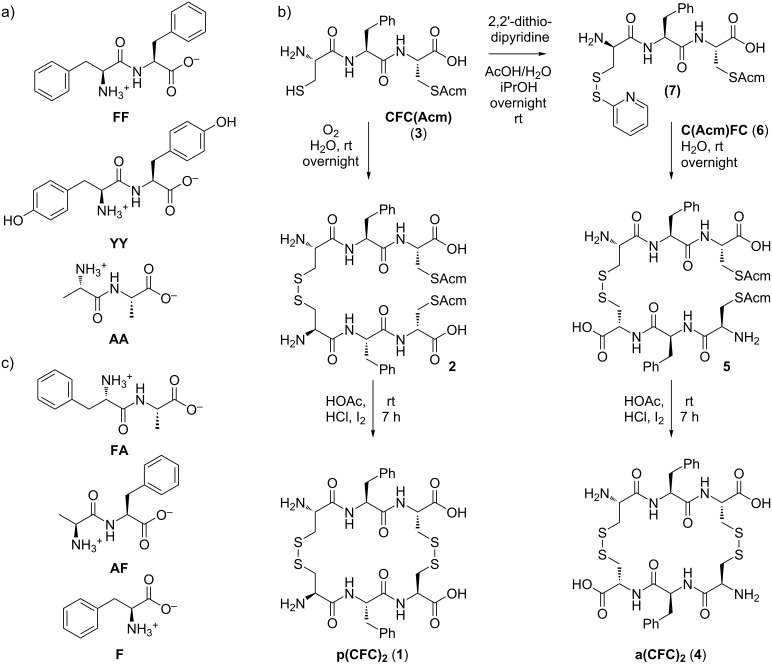
a) Synthesis of the parallel and antiparallel isomers **p(CFC)_2_** and **a(CFC)_2_**_._ b) Templates **FF**. **YY** and **AA**. c) Dipeptides **FA**, **AF** and amino acid **F** used for comparison.

Upon addition of certain templates, notable changes were found in the chromatograms of the **CFC** dimers in comparison to the reference (Figure 2a, full chromatograms Figures S2–S4, Supporting Information File 1). Amplification of both peak areas is observed for all templates, which indicates an interaction between the dipeptides and the cyclic tripeptide dimers. For the second peak the change is most significant for **FF**, followed by **YY** and **AA**, respectively (Figure 2b, Scheme 2a). For the first peak, however, the effect of **FF** is the weakest. This could indicate a selectivity of the binding to the isomer of the second peak, though it is more likely that ionization at the first peak is suppressed by co-elution with the **FF** template. This is a not an issue for the **YY** containing sample as the template peak maximum lays between the first and second tripeptide dimer peak. We therefore concluded that the binding affinities of the receptor template pairs should be **FF** > **YY** > **AA**.

**Figure 3:**
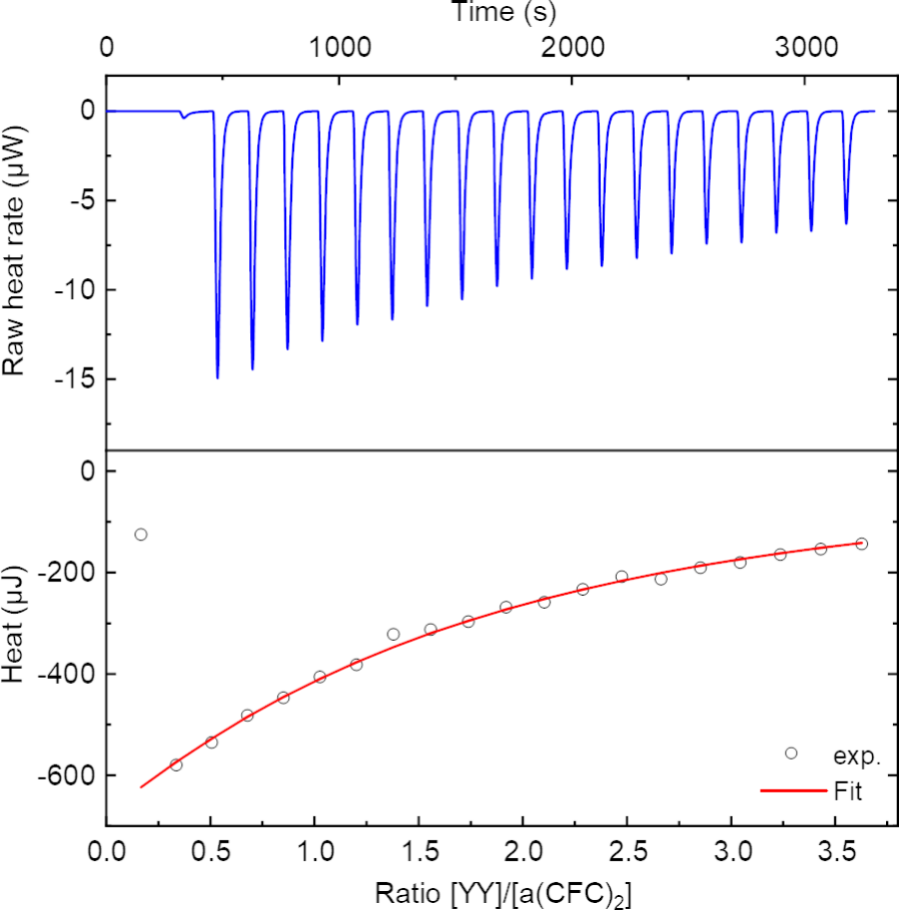
ITC of **YY** (30 mM) to **a(CFC)_2_** (1.5 mM) in phosphate buffer (pH 7.4, 100 mM).

In order to conclusively to test for binding behavior and selectivity, both isomers **a(CFC)_2_** and **p(CFC)_2_** were synthesized using a suitable protecting group strategy (Scheme 2b). One of the Trt protection groups was replaced by an acetamidomethyl (Acm) group (**CFC(Acm)**), which is stable under the cleaving conditions of the SPPS. Thus one cysteine moiety is left deprotected and can be addressed selectively either by dimerization with another **CFC(Acm)** or with inversely substituted tripeptide (**C(Acm)FC**). These linear peptide dimers were subsequently cyclized by oxidative cleavage of the Acm groups to give **a(CFC)_2_** and **p(CFC)_2_**, respectively. The binding affinities and thermodynamic data were quantified by ITC measurements (Table 1, Figure 3 and Figures S5–S9, Supporting Information File 1).

**Table 1:** Binding behavior of several peptides (30 mM) towards **p(CFC)_2_** and **a(CFC)_2_** (1.5 mM)**_._** Measured by ITC in phosphate buffer (pH 7.4, 100 mM). Data fitted with a 1:1 model.

tripeptide dimer	dipeptide	*K* [M^−1^]	Δ*G* [kJ/mol]	Δ*H* [kJ/mol]	Δ*S* [J/mol·K]

**a(CFC)_2_** **p(CFC)_2_**	**YY** **YY**	297 285	−14.1 −14.0	−28.3 −30.6	−47.6 −55.8
**a(CFC)_2_** **p(CFC)_2_**	**FF** **FF**	702^a^ 229^a^	−16.2^a^ −13.5^a^	−2.1^a^ −4.0^a^	47.4^a^ 31.7^a^
**a(CFC)_2_** **p(CFC)_2_**	**AA** **AA**	75 61	−10.7 −10.2	−4.6 −7.4	20.5 9.4
**a(CFC)_2_**	**AF**	100	−11.4	−6.9	15.0
**a(CFC)_2_**	**FA**	108	−11.6	−13.0	−4.6
**a(CFC)_2_**	**F**	52	−9.8	−0.9	30.00

^a^Poor solubility made it necessary to use lower concentrations. **FF** (5 mM) to tripeptide dimers (250 µM). Therefore, values contain uncertainty due to low heat rates.

All investigated interactions show a negative enthalpy and are therefore exothermal. Though the magnitude of these values differs based on the dipeptide. The binding constants of **YY** to **a(CFC)_2_** and **p(CFC)_2_** are very similar and no selectivity is observed.

In the interaction of **FF** with **a(CFC)_2_** and **p(CFC)_2_** the enthalpy is very low, which is cause for a poor signal-to-noise ratio. Other values for these particular interactions should therefore be met with some caution. **FF** seems to bind stronger to **a(CFC)_2_** than to **p(CFC)_2_**, though again the values show some uncertainty and real values of the binding constants are likely in the same order of magnitude as for **YY**. Unlike for the interaction with **FF**, the entropy change of interaction with **YY** appears to be highly positive, which might be due to hydrophobic interactions. We do not attribute this behavior to a fundamentally different mode interaction compared to **FF** though, as both dipeptides behave very similar in the following NMR experiments. The effect may be attributed to the intermolecular interaction of the dipeptides with themselves. For example, **FF** is a popular self-assembly motif, which also explains its poor solubility (≈5 mM) [23]. Hence the thermodynamic data depicts not only the association of **a(CFC)_2_**/**p(CFC)_2_** towards **FF**, but also the dissociation of **FF** assemblies. The aromatic side groups of **FF** and **YY** seem to play an important role in the binding, since **AA** binds significantly weaker. Each of the aromatic units contributes to the binding to a roughly equal amount, as is shown by a comparison with **AF** and **FA** (Scheme 2c), which both bind equally strong. Notably, adding one phenyl function yields a smaller increase in binding affinity than adding a second one, pointing towards a cooperative contribution of both phenyl rings. Pure **F** is the weakest tested binder, which hints at the selectivity towards dipeptides.

**Figure 4:**
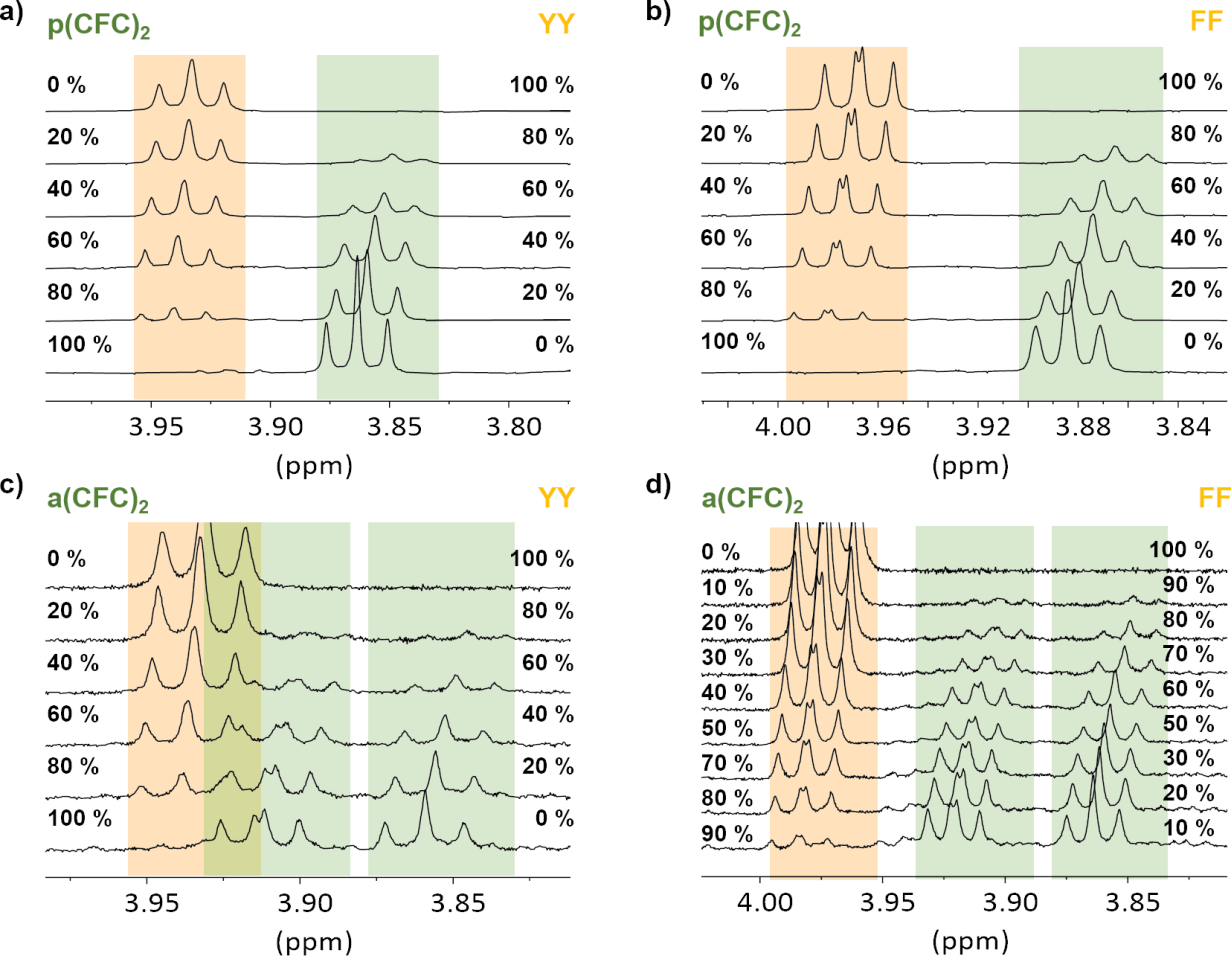
Continuously varied NMR measurements of a) **p(CFC)_2_** to **YY** b) **p(CFC)_2_** to **FF** c) **a(CFC)_2_** to **YY** d) **a(CFC)_2_** to **FF**.

**Figure 5:**
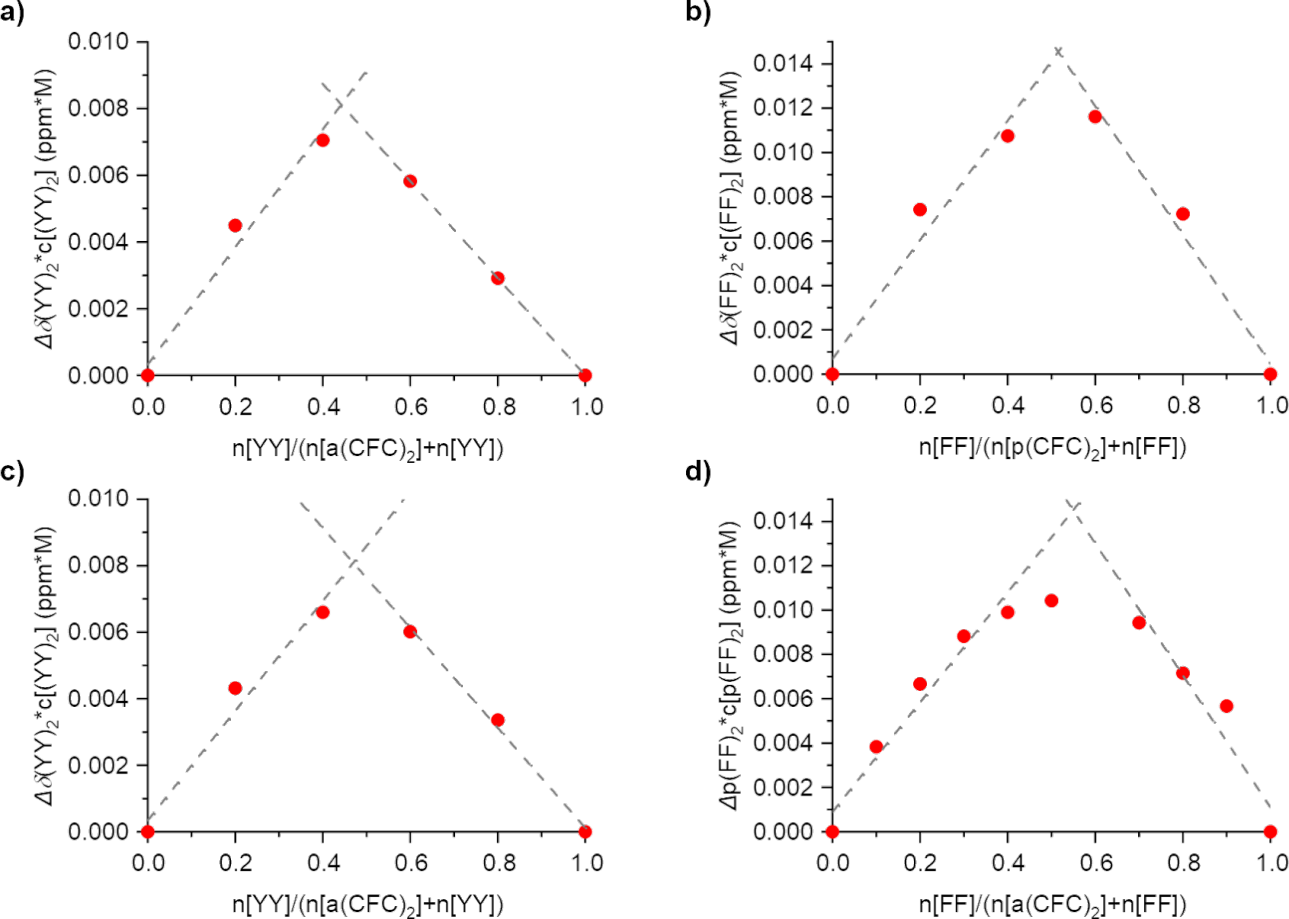
Job plots derived from the continuously varied NMR measurements of a) **p(CFC)_2_** to **YY** b) **p(CFC)_2_** to **FF** c) **a(CFC)_2_** to **YY** d) **a(CFC)_2_** to **FF**.

Next we used NMR spectroscopy to further characterize the binding behavior. Initial titrations of **YY** or **FF** to **a(CFC)_2_** or **p(CFC)_2_** hinted at formation of H-bonds due to shifts of protons at α-carbon atoms between δ = 3.85 to 4.00. However, no binding constants could be determined from those measurements as even in a 10-fold excess the system showed no signs of saturation (Figure S10, Supporting Information File 1). This behavior does not contradict the ITC measurements and can be attributed to the kinetic isotope effect. Although, D-bonding is generally stronger than H-bonding, its contribution to the peptide–peptide binding can be weakened by even stronger competitive binding to the D_2_O solvent [24]. The shifting signals could be used to obtain Job plots which indicate 1-to-1 complexes for all combinations of **YY** or **FF** with **a(CFC)_2_** or **p(CFC)_2_** (Figure 4 and Figure 5), since the maxima are around x ≈ 0.5. Their profiles show shallow curves instead of sharp transitions, which is in agreement with the apparently weaker interaction in D_2_O [25].

## Conclusion

In summary, we demonstrated that artificial peptide receptors can emerge from a peptide-based DCL under competitive conditions in water. In agreement with our previous works on carbohydrate recognition, this supports our initial assumption that dynamic peptides, due to their similarity towards enzymes, can bind a broad scope of biomolecules. ITC measurements suggest that the cyclic tripeptide dimers **a(CFC)_2_** and **p(CFC)_2_** are stronger binders for the aromatic dipeptides **FF** and **YY** (*K* ≈ 229–702 M^−1^), then for the non aromatic **AA**, which is bound less efficiently with a factor of about 3–10 times weaker (*K* ≈ 65–71 M^−1^).

## Experimental

### Preparation of the peptide libraries

Stock solutions of each CXC building blocks (7.5 mM each) were prepared in NH_4_CO_3_ buffer (pH 7.8, 100 mM). 50 µL stock solution of each was given to a 1.5 mL vial. Another 100 µL of a template stock solution (90 mM) was added. For a reference sample the template solution was substituted for buffer. Small magnetic stirring bars were added, and the vials were covered by a layer of perforated Parafilm to slow down drying due to fast air exchange. After stirring overnight (100 rpm), the samples were acidified with a 5% solution of formic acid in water. The samples were diluted 1:10 with water and measured with HPLC–MS setup I.

### HPLC–MS measurements

The HPLC setup was based on a Shimadzu system and consisted of a CBM-20A controller, a SIL-HTA auto sampler, two 10ADVP pumps, a DGU-14A degasser, a CTO-10AVP column oven and an SPD-10AVP variable wavelength detector. The setup was coupled to a micrOTOF time of flight mass analyzer (Bruker) and controlled by Compass HyStar (Bruker) version 3.2 and microTOFControl (Bruker) version 3.0. Measurement data was analyzed using Compass DataAnalysis version 5.0 R1 (Bruker).

The column oven was set to 40 °C. A SeQunant ZIC-HILIC (Merk KGaA, 150 × 2.1 mm, particle size 3.5 µM, pore size 200 Å) with a SeQunant ZIC-HILIC (Merk KGaA, 20 × 2.1 mm, particle size 5 µM, pore size 200 Å) precolumn was used as stationary phase. The mobile phase was a mixture of water (0.1 % NH_4_FA, pH 3.2) and acetonitrile. The injection volume was 5 µL. The following gradient was used with a flow rate of 150 µL/min (Table 2).

**Table 2:** Solvent gradients for HPLC.

time (min)	0	15	17	19	20	22	23	49

water (%)	45	51	60	60	40	40	45	45
ACN (%)	55	49	40	40	60	60	55	55

### ITC measurements

For determination of binding constants ITC was measured in phosphate buffered solutions (pH 7.4, 100 mM). Concentration were used as noted. In general, the titrant concentration was 20 times the concentration of the other peptide. ITC was performed on a Nano ITC low volume titration calorimeter (TA Instruments) with a cell volume of 170 µL. The program used 20 injections of 2.5 µL each in an interval of 2.5 min and a stirring rate of 350 rpm. The device was operated using ITCRun version 2.1.7.0 (TA Instruments). For each experiment a correction for the dilution was measured by titrating the titrant to pure solvent. For initial analysis of the heat rates NanoAnalyze version 3.8.0 (TA Instruments) was used. Data fits and thermodynamic data was obtained using a numerical approximation using Microsoft Excel.

### NMR experiments

For the Job plots 3 mM stock solutions of each component in deuterated buffer were mixed in different ratios with an interval of 10% or 20%. Spectra were recorded on a DD2 500 (Agilent) or a DD2 600 (Agilent) spectrometer. Small amounts of DMSO (83 nM) were used as reference (δ = 2.62 ppm). Variable temperature ^1^H NMR of **a(CFC)_2_** was recorded in pure D_2_O.

Deuterated buffer was prepared by the following procedure. Regular non-deuterated NaHPO_4_·2H_2_O (156 mg, 1 mmol) was dissolved in D_2_O (1 mL) and afterwards dried in vacuo. This step was repeated for a total number of three times. The salt was dissolved again in D_2_O (10 mL) and the pD was adjusted with NaOD (40 wt % in D_2_O) using a pH-calibrated glass electrode and the following relation [26].


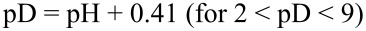


## Supporting Information

File 1 Synthesis, additional data, and NMR spectra.

## References

[1] Cram D J, Cram J M (1978). Acc Chem Res.

[2] Famulok M, Jeong K-S, Deslongchamps G, Rebek J (1991). Angew Chem, Int Ed Engl.

[3] Hossain M A, Schneider H-J (1998). J Am Chem Soc.

[4] Schmuck C, Wich P (2006). Angew Chem, Int Ed.

[5] Schmuck C, Heil M (2006). Chem – Eur J.

[6] Schmuck C, Rupprecht D, Wienand W (2006). Chem – Eur J.

[7] Moure A, Luis S V, Alfonso I (2012). Chem – Eur J.

[8] Larsen D, Beeren S R (2019). Chem Sci.

[9] Klein J M, Clegg J K, Saggiomo V, Reck L, Lüning U, Sanders J K M (2012). Dalton Trans.

[10] Chung M-K, Severin K, Lee S J, Waters M L, Gagné M R (2011). Chem Sci.

[11] Furlan R L E, Ng Y-F, Otto S, Sanders J K M (2001). J Am Chem Soc.

[12] Otto S, Furlan R L E, Sanders J K M (2002). Science.

[13] Corbett P T, Tong L H, Sanders J K M, Otto S (2005). J Am Chem Soc.

[14] Vial L, Ludlow R F, Leclaire J, Pérez-Fernández R, Otto S (2006). J Am Chem Soc.

[15] Ghosh S, Ingerman L A, Frye A G, Lee S J, Gagné M R, Waters M L (2010). Org Lett.

[16] Darbre T, Reymond J-L (2006). Acc Chem Res.

[17] Hioki H, Still W C (1998). J Org Chem.

[18] Buryak A, Severin K (2005). Angew Chem, Int Ed.

[19] Rauschenberg M, Bomke S, Karst U, Ravoo B J (2010). Angew Chem, Int Ed.

[20] Rauschenberg M, Bandaru S, Waller M P, Ravoo B J (2014). Chem – Eur J.

[21] Otremba T, Ravoo B J (2016). ChemistrySelect.

[22] Yoshida T (2004). J Biochem Biophys Methods.

[23] Reches M, Gazit E (2006). Nat Nanotechnol.

[24] Bowers P M, Klevit R E (1996). Nat Struct Mol Biol.

[25] Renny J S, Tomasevich L L, Tallmadge E H, Collum D B (2013). Angew Chem, Int Ed.

[26] Covington A K, Paabo M, Robinson R A, Bates R G (1968). Anal Chem (Washington, DC, U S).

