# Supplementary material for: A dynamic combinatorial library for biomimetic recognition of dipeptides in water
Source: Beilstein J Org Chem. 2020 Jul 2;16:1588–95. doi: 10.3762/bjoc.16.131 (PMC7356556; doi:10.3762/bjoc.16.131)
Supplement: File 1 — Synthesis, additional data, and NMR spectra. [file Beilstein_J_Org_Chem-16-1588-s001.pdf]

BEILSTEIN JOURNAL OF ORGANIC CHEMISTRY

Supporting Information

for

A dynamic combinatorial library for biomimetic recognition of dipeptides in water

Florian Klepel and Bart Jan Ravoo

Beilstein J. Org. Chem. **2020,** *16,* 1588–1595. doi:10.3762/bjoc.16.131

Synthesis, additional data, and NMR spectra

License and Terms: This is a supporting information file under the terms of the Creative Commons Attribution License (http://creativecommons.org/licenses/by/4.0). Please note that the reuse, redistribution and reproduction in particular requires that the authors and source are credited.
The license is subject to the *Beilstein Journal of Organic Chemistry* terms and conditions: (https://www.beilstein-journals.org/bjoc)

1 Synthesis

1.1 Solid-phase peptide synthesis (SPPS)

Peptide synthesis was performed according to standard procedures described in literature.[1,2]

1.1.1 Loading of the resin (PS 1)

Loading of the resin was performed under an atmosphere of argon. The according amino acid (1.5 equiv relative to resin loading) was dissolved in dry DCM and a small amount of DMF (synthesis grade) and was added to 2-chlorotrityl-resin (1.6 mmol/g resin). After addition of DIPEA (2 equiv relative to resin loading) the mixture was agitated by a stream of argon for 5 min. Following another addition of DIPEA (3 equiv relative to resin loading), agitation was continued for 1 h. The remaining reactive groups were quenched with MeOH p.a. (1 mL/g resin) for 15 min. After filtration of the resin it was washed consecutively with DCM p.a. (3 × 20 mL), DMF p.a. (3 × 20 mL), DCM p.a. (3 × 20 mL) and MeOH p.a. (3 × 20 mL) and was dried until weight constant was reached.

Chain elongation was done either manually (PS 2A) or automated (PS 2B). Which method was used is mentioned in the paragraph of the corresponding peptide.

1.1.2 Manual synthesis (PS 2A)

Dry beads prepared as described in PS 1 were swollen in DMF p.a. for 45 min by shaking the reaction vessel. After sucking of the DMF p.a. the Fmoc-group was cleaved with 20% piperidine in DMF for 20 min. The resin was washed with DMF p.a. (4 times) and alternately with DCM p.a. (3 times) and isopropanol p.a. (3 times). The success of cleavage was controlled by the Kaiser test. Equal amounts of 5% ninhydrin in EtOH, 80% phenol in EtOH and 0.001 M KCN in pyridine were mixed with a few beads and heated for 1 min to 100 °C. A blue color indicated free amine functions on the resin.

The coupling step was performed by suspending the resin in a solution of the according Fmoc-protected amino acid (3 equiv relative to resin loading) and Oxyma pure (4.0 equiv) in DMF (SPPS grade). After addition of DIC (3.0 equiv), the suspension was shaken for 2 h and then the solvent is sucked off. Washing of the beads with DMF (SPPS grade, 4 times) was followed by the Kaiser test which should not result in a color change if the coupling was successful. The deprotection and coupling steps were continued until the desired peptide sequence was obtained.

1.1.3 Automated synthesis (PS 2B)

Automated SPPS was performed on a CS136 Peptide synthesizer (CSBio) using the CSBio Peptides Synthesizer software version 3.0.0.0 (CSBio). The loaded resin was preswollen by shaking for 5 min in DMF. For Fmoc-deprotection a solution of 20% piperidine in DMF was added and the dispersion was mixed for 4 min. After rinsing the cleavage procedure was repeated with a mixing time of 19 min. Subsequently the resin was washed seven times with DMF. For peptide coupling an amino acid solution was loaded in the reaction vessel. This was followed by addition of 3 equiv of a 0.4 M HBTU/HOBt solution and 4.5 equiv of a 0.6 M DIPEA solution. The reaction vessel was mixed for 2.5 h. The deprotection and coupling steps were continued until the desired peptide sequence was obtained.

1.1.4 Cleavage from resin and removal from permanent protecting groups (PS 3)

Cleavage of the resin and removal of permanent protection groups was performed according to common literature procedures. Prior to splitting of the peptide from the resin, the *N*-terminal Fmoc-group has to be removed by treatment with 20% piperidine in DMF for 20 min. After washing with DMF p.a. (4 times) and alternately with DCM (2 times) and isopropanol p.a. (2 times), the beads were suspended in a solution of TFA/H_2O/EDT/TIS = 94:2.5:2.5:1 and stirred for 8 h at room temperature. After

filtration, the beads were washed with TFA (5 times) and the filtrate was concentrated until the peptide started to precipitate. Addition of cold Et_2O resulted in complete precipitation and the suspension was kept overnight in the freezer. The peptide was collected by filtration, washed with Et_2O and dried in high vacuum to yield the peptides as white, hygroscopic crude products which were stored under argon.

1.2 Tyr-Tyr (YY)

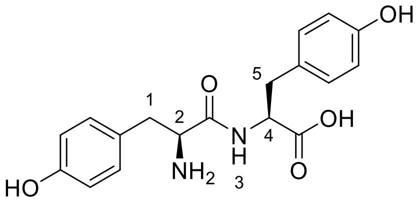

Tyr-Tyr was synthesized according to the general procedure for manual SPPS and purified by preparative HPLC.

Yield: 46.1 mg (134 µmol, 10%).

HR-MS: $[M+H]^+$: *m*/z = 345.1440 (calc. 345.1456).

^{1}H-NMR: (300 MHz, DMSO-*d*$_6$) *δ* = 9.37 (s, 1H, Ar-O*H*), 9.28 (s, 1H, Ar-O*H*), 8.78 (d, *J* = 7.9 Hz, 1H, 5-N*H*), 7.12 – 6.97 (m, 4H, Ar-*H*), 6.74 – 6.63 (m, 4H, Ar-*H*), 4.43 (td, *J* = 8.1, 5.1 Hz, 1H, 4-C*H*), 3.92 (s, 1H, 2-C*H*), 3.00 (m, 2H, 1-C*H*$_2$), 2.89 – 2.69 (m, 2H, 5-C*H*$_2$).

1.3 Phe-Phe (FF)

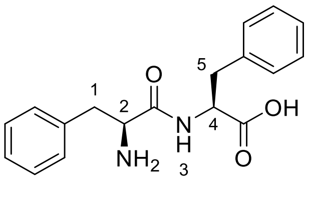

Phe-Phe was synthesized according to the general procedure for manual SPPS and purified by automated reverse phase chromatrography using the gradient below.

Time (min)	0	15	20
Water (%)	95	20	20
ACN (%)	5	80	80
ID C18-WP 24 g column, 32 mL/min			

Yield: 229 mg (732 µmol, 53%).

HR-MS: $[M+H]^+$: m/z = 313.1545 (calc. 313.1547).

$[M+Na]^+$: m/z = 335.1359 (calc. 335.1366).

^{1}H-NMR: (500 MHz, DMSO-d_6) δ = 8.90 (d, J = 7.8 Hz, 1H, 3-NH), 7.36 – 7.18 (m, 10H, Ar-H), 4.52 (td, Hz = 8.2, 5.3 Hz, 1H, 4-CH), 4.06 (dd, J = 8.2, 4.9 Hz, 1H, 2-CH), 3.17 – 3.07 (m, 2H, 1-CH_2), 2.95 (td, J = 13.8, 8.3 Hz, 2H, 5-CH_2).

^{13}C-NMR: (126 MHz, DMSO-d_6) δ = 172.2, 168.2, 137.2, 134.7, 129.6, 129.2, 128.5, 128.3, 127.2, 126.6, 53.8, 53.1, 36.9, 36.7.

1.4 Ala-Phe (AF)

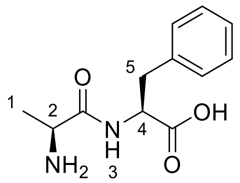

Ala-Phe was synthesized according to the general procedure for manual SPPS and purified by automated reverse phase chromatrography using the gradient below.

Time (min)	0	15	20
Water (%)	95	20	20
ACN (%)	5	80	80
ID C18-WP 24 g column, 32 mL/min			

Yield: 23.6 mg (100 µmol, 7%).

HR-MS: $[M+H]^+$: m/z = 237.1230 (calc. 237.1234).

$[M+Na]^+$: m/z = 259.1048 (calc. 259.1059).

^{1}H-NMR: (400 MHz, DMSO-d_6) δ = 8.70 (d, J = 7.9 Hz, 1H, 3-NH), 7.34 – 7.16 (m, 5H, Ar-H), 4.46 (ddd, J = 9.4, 7.8, 4.7 Hz, 1H, 4-CH), 3.81 (s, 1H, 2-CH), 3.11 (dd, J = 14.0, 4.8 Hz, 1H, 5-CH_2), 2.92 (dd, J = 14.0 Hz, 9.5, 1H, 5-CH_2), 1.33 (d, J = 7.0 Hz, 3H, 1-CH_3).

1.5 Phe-Ala (FA)

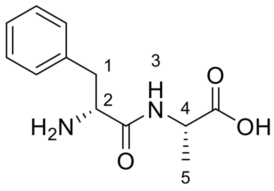

Phe-Ala was synthesized according to the general procedure for manual SPPS and purified by automated reverse phase chromatrography using the gradient below.

Time (min)	0	15	20
Water (%)	95	20	20
ACN (%)	5	80	80
ID C18-WP 24 g column, 32 mL/min			

Yield: 14 mg (60 µmol, 4%).

HR-MS: $[M+H]^+$: *m*/z = 237.1234 (calc. 237.1234).

$[M+Na]^+$: *m*/z = 259.1049 (calc. 259.1059).

^{1}H-NMR: (400 MHz, DMSO-d_6) δ = 8.83 (d, J = 7.1 Hz, 1H, 3-N*H*), 7.39 – 7.20 (m, 5H, Ar-*H*), 4.27 (p, J = 7.2 Hz, 1H, 4-C*H*), 4.02 (dd, J = 8.0, 5.3 Hz, 1H, 2-C*H*), 3.17 – 3.07 (m, 1H, 1-CH_2), 2.92 (dd, J = 14.1, 8.0 Hz, 1H, 1-CH_2), 1.32 (d, J = 7.3 Hz, 3H, 5-CH_3).

1.6 Cys-Phe-Cys(Acm) – CFC(Acm) – (3)

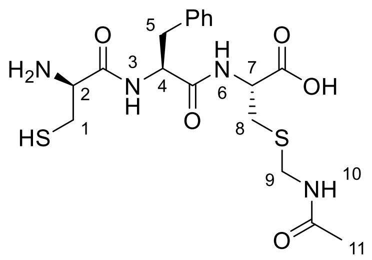

Cys-Phe-Cys(Acm) was synthesized according to the general procedure for automated SPPS and purified by automated reverse phase chromatrography using the gradient below.

Time (min)	0	15	20
Water (%)	95	20	20
ACN (%)	5	80	80
ID C18-WP 24 g column, 32 mL/min			

Yield: 485 mg, 1.09 mmol, 55%.

HR-MS: $[M+H]^+$: *m*/z = 443.1412 (calc. 443.1417).

$[M+Na]^+$: *m*/z = 465.1231 (calc. 465.1237).

^{1}H-NMR: (400 MHz, DMSO-d_6) δ = 8.67 (t, J = 9.7, 8.0 Hz, 2H, 3-/10-NH), 8.56 (t, J = 6.3 Hz, 1H; 6-NH), 7.34 – 7.23 (m, 4H, *o*-/*m*-Ar-H), 7.23 – 7.17 (m, 1H, *p*-Ar-H), 4.64 (ddd, J = 10.1, 8.1, 3.9 Hz, 1H, 7-CH), 4.45 (td, J = 8.4, 4.7 Hz, 1H, 4-CH), 4.24 (dd, J = 6.4, 5.0 Hz, 2H, 9-CH_2), 3.96 (dd, J = 5.9, 4.2 Hz, 1H, 2-CH), 3.15 – 2.75 (m, 6H, 1-/5-/8-CH_2), 1.85 (s, 3H, 11-CH_3).

^{13}C-NMR: (101 MHz, DMSO-d_6) δ = 171.8, 171.0, 169.6, 166.9, 137.5, 129.2, 128.2, 126.5, 54.4, 53.7, 52.5, 40.6, 37.2, 31.9, 25.6, 22.6.

1.7 Parallel Cys(Acm)-Phe-Cys-S-S-Cys-Phe-Cys(Acm) – (2)

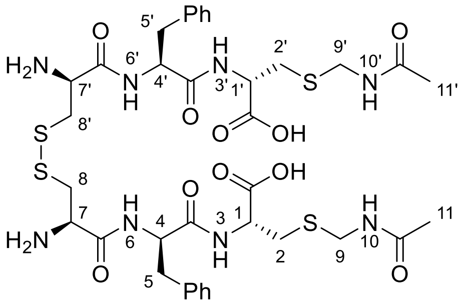

Cys-Phe-Cys(Acm) (**3**) (230mg, 678 µmol, 1.0 equiv) was dissolved in water. The solution was stirred for 17 h at room temperature. Afterwards the solvent was removed by freeze drying to yield the product as a white solid.

Time (min)	0	16.5	22
Water (%)	95	20	20
ACN (%)	5	80	80
ID C18-WP 24 g column, 20 mL/min			

Yield: 230 mg, 260 µmol, 77%.

HR-MS: $[M+H]^+$: m/z = 443.14189 (calc. 443.14174).

^{1}H-NMR: (400 MHz, DMSO-d_6) δ = 8.68 (t, J = 6.5 Hz, 2H, NH), 8.57 (d, J = 7.9 Hz, 2H, NH), 8.42 (d, J = 8.1 Hz, 2H, NH), 7.38 – 7.15 (m, 10H, ArH), 4.66 (td, J = 8.9, 4.1 Hz, 2H, 1-CH), 4.52 – 4.37 (m, 4H, 9-CH_2), 4.16 (dd, J = 13.7, 5.7 Hz, 2H, 4-CH_4), 4.01 (dd, J = 9.2, 3.6 Hz, 2H, 7-CH), 3.07 (m, 4H, CH_2), 2.91 – 2.71 (m, 8H, CH_2), 1.91 (s, 6H, 11-CH_3).

1.8 Parallel (Cys-Phe-Cys)$_2$ – p(CFC)$_2$ – (1)

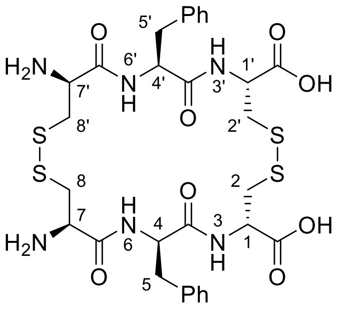

The synthesis was performed analogous to literature with slight modifications.[3]A 17 mM iodine solution in HOAc (29 mL) was dropped to a solution of parallel Cys(Acm)-Phe-Cys-S-S-Cys-Phe-Cys(Acm) (**2**) (224 mg, 254 µmol, 1.0 equiv) in HOAc (450 mL) and HCl (3.3 mL). The reaction was stirred for 7 h. Afterwards the reaction mixture was diluted with water and extracted several times with pentane to remove iodine. The aqueous phase was dried in vacuo and purified by automated reverse phase flash chromatography.

Time (min)	0	10	15	20
Water (%)	85	85	0	0
ACN (%)	2	30	80	80
ID C18-WP 24 g column, 32 mL/min				

Yield: 81 mg, 109 µmol, 43%.

HR-MS: $[M+H]^+$: m/z = 739.17113 (calc. 739.17067).

$[M+Na]^+$: m/z = 761.15299 (calc. 761.15262).

^{1}H-NMR: (500 MHz, DMSO-d_6) δ = 9.09 (s, 2H, N*H*), 8.50 (s, 2H, N*H*), 7.34 – 7.13 (m, 10H, Ar*H*), 4.65 (q, *J* = 7.4 Hz, 2H, C*H*), 4.62 – 4.55 (m, 3H, C*H*), 4.10 (t, *J* = 6.2 Hz, 2H, C*H*), 3.19 – 2.72 (m, 12H, CH_2).

^{13}C-NMR: (126 MHz, DMSO-d_6) δ = 171.7, 170.1, 166.7, 137.1, 129.2, 128.2, 126.4, 54.0, 50.8, 50.5, 38.2, 38.1, 37.7.

1.9 Cys(pyridin-2-ylthio)-Phe-Cys(Acm) – (7)

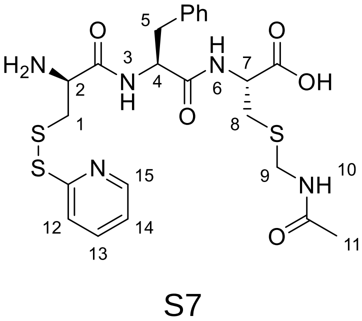

The synthesis was performed according to literature with some modifications.[3] Cys-Phe-Cys(Acm) (**3**) (739 mg, 1.67 mmol, 1.0 equiv) dissolved in AcOH (28 mL). To this solution, 2,2'-dithiodipyridine (368 mg, 1.67 mmol, 1.0 equiv) in iPrOH (320 mL) was added and stirred at room temperature for 18 h. The solvent was removed, and the residue was dissolved in a small amount of TFA. The raw product was obtained by precipitation with cold Et_2O and was purified by reverse phase flash chromatography.

Time (min)	0	15	20
Water (%)	87	20	20
ACN (%)	13	80	80
ID C18-WP 24 g column, 32 mL/min			

Yield: 610 mg, 1.11 mmol, 66%.

HR-MS: $[M+H]^+$: m/z = 552.1390 (calc. 552.1404).

$[M+Na]^+$: m/z = 574.1209 (calc. 574.1223).

^{1}H-NMR: (400 MHz, DMSO-d_6) δ = 8.87 (dd, J = 23.6, 8.1 Hz, 1H, Ar-H or NH), 8.75 – 8.37 (m, 4H, Ar-H or NH), 7.91 – 7.58 (m, 1H, Ar-H or NH), 7.38 – 7.15 (m, 6H, Ar-H or NH), 4.68 (dtd, J = 15.3, 8.8, 4.2 Hz, 1H, 7-CH), 4.49 – 4.38 (m, 1H, 4-CH), 4.31 – 4.15 (m, 2H, 9-CH_2), 4.04 (ddd, J = 33.1, 9.0, 4.3 Hz, 1H, 2-CH), 3.17 – 2.74 (m, 6H, 1-/5-/8-CH_2), 1.88 – 1.78 (m, 3H, 11-CH_3).

^{13}C-NMR: (101 MHz, DMSO-d_6) δ = 171.8, 171.8, 170.7, 170.5, 169.7, 169.6, 167.1, 166.7, 157.7, 149.9, 149.8, 138.2, 138.0, 137.3, 129.3, 128.2, 128.2, 126.5, 121.8, 120.6, 119.6, 54.3, 52.6, 52.4, 51.3, 40.7, 40.6, 37.4, 31.9, 22.6.

1.10 Cys(Acm)-Phe-Cys – C(Acm)FC – (6)

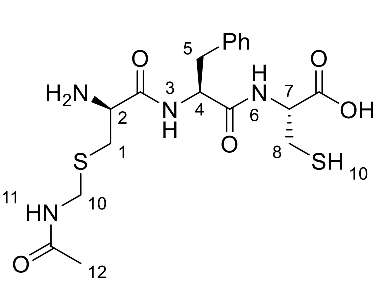

Cys(Acm)-Phe-Cys was synthesized according to the general procedure for automated SPPS and purified by automated reverse phase chromatrography using the gradient below.

Time (min)	0	9	12	15
Water (%)	85	85	0	0
ACN (%)	15	15	100	100
ID C18-WP 24 g column, 40 mL/min				

Yield: 220 mg, 497 µmol, 25%.

HR-MS: $[M+H]^+$: m/z = 443.1415 (calc. 443.1417).

$[M+Na]^+$: m/z = 465.1238 (calc. 465.1237).

$[M-H]^-$: m/z = 441.1265 (calc. 441.1272).

^{1}H-NMR: (400 MHz, DMSO-d_6) δ = 8.69 (t, J = 6.4 Hz, 1H, 11-N*H*), 8.56 (d, J = 7.9 Hz, 1H, 6-N*H*), 8.46 (d, J = 8.0 Hz, 1H, 3-N*H*), 7.36 – 7.25 (m, 4H, *o-/m-*Ar-*H*), 7.24 – 7.18 (m, 1H, *p*-Ar-*H*), 4.65 (ddd, J = 9.7, 8.0, 4.3 Hz, 1H, 7-C*H*), 4.49 – 4.38 (m, 2H, 10-CH_2), 4.16 (dd, J = 13.7, 5.8 Hz, 1H, 4-CH_2), 4.02 (dd, J = 9.0, 3.8 Hz, 1H, 2-CH_2), 3.15 – 2.70 (m, 6H, 8-/5-/1-CH_2), 2.40 (t, J = 8.5 Hz, 1H, 10-S*H*), 1.91 (s, 3H, 12-CH_3).

^{13}C-NMR: (101 MHz, DMSO-d_6) δ = 171.3, 170.7, 170.7, 167.5, 137.3, 129.3, 128.2, 126.5, 54.5, 54.3, 51.6, 37.4, 35.8, 31.7, 30.8, 25.6, 22.6.

1.11 Antiparallel Cys(Acm)-Phe-Cys-S-S-Cys-Phe-Cys(Acm) – (5)

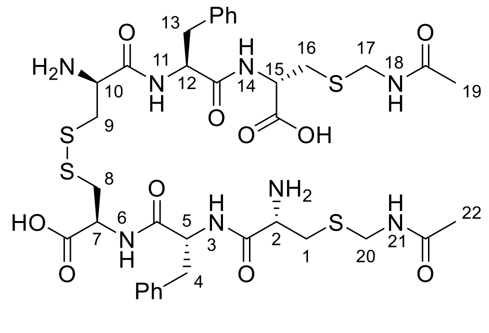

The synthesis was performed analogous to literature with slight modifications.[3] Solutions of Cys(pyridin-2-ylthio)-Phe-Cys(Acm) (287 mg, 521 µmol, 1.1 equiv) in water (80 mL) and Cys(Acm)-Phe-Cys (**6**) (210 mg, 473 µmol, 1.0 equiv) in water (120 mL) were adjusted to pH 8.5 with NH_4OH and subsequently combined. After 16 h of

stirring the reaction was stopped by addition of AcOH (2 mL). The solvent was removed *in vacuo* to give a yellow oil. The crude product was purified by automated reverse phase chromatography and the pure product was obtained as a white solid.

Time (min)	0	16.5	22
Water (%)	95	20	20
ACN (%)	5	80	80
ID C18-WP 24 g column, 20 mL/min			

Yield: 610 mg, 1.11 mmol, 66%.

HR-MS: $[M+H]^+$: *m*/z = 552.1390 (calc. 552.1404).

$[M+Na]^+$: *m*/z = 574.1209 (calc. 574.1223).

^{1}H-NMR: (300 MHz, $CDCl_3$) δ = 8.85 (d, *J* = 8.0 Hz, 1H, N*H*), 8.78 – 8.45 (m, 4H, N*H*), 7.37 – 7.15 (m, 10H, Ar-*H*), 4.74 – 3.98 (m, 10H, *α*-C*H* and 17-/20-CH_2), 3.36 – 2.69 (m, 12H, 1-/4-/7-/9-/13-/16-CH_2), 1.91 (s, 3H, 19- or 22-CH_3), 1.85 (s, 3H, 19- or 22-CH_3).

^{13}C-NMR: (75 MHz, $CDCl_3$) δ = 177.0, 175.9, 175.9, 175.0, 174.9, 172.8, 172.3, 172.1, 163.7, 163.2, 142.6, 142.5, 134.6, 134.5, 133.4, 133.3, 131.9, 124.3, 120.4, 59.6, 59.6, 59.5, 59.4, 57.8, 57.7, 56.7, 45.9, 45.9, 42.6, 37.1, 37.0, 36.8, 27.9, 27.8.

1.12 Antiparallel (Cys-Phe-Cys)$_2$ – a(CFC)$_2$ – (4)

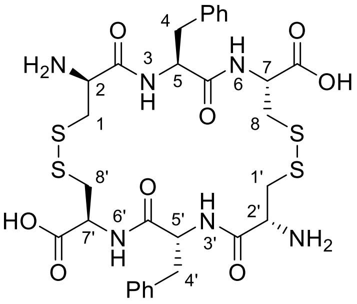

The synthesis was performed analogous to literature with slight modifications.[3] A 20 mM Iodine solution in HOAc (18 mL) was dropped to a solution of Cys(Acm)-Phe-Cys-S-S-Cys-Phe-Cys(Acm) **(5)** (320 mg, 363 µmol, 1 equiv) in HOAc (290 mL) and HCl (2.06 mL). The reaction was stirred for 7 h. Afterwards the reaction mixture was diluted with water and extracted several times with pentane to remove Iodine. The

aqueous phase was dried in vacuo and purified by automated reverse phase flash chromatography.

Time (min)	0	15	20
Water (%)	95	20	20
ACN (%)	5	80	80
ID C18-WP 24 g column, 32 mL/min			

Yield: 238 mg, 321 µmol, 89%.

HR-MS: $[M+2H]^{2+}$: m/z = 370.08863 (calc. 370.08897).

$[M+H]^{+}$: m/z = 739.17053 (calc. 739.17067).

^{1}H-NMR: (500 MHz, DMSO-d_6) δ = 9.06 (s, 1H, N*H*), 8.91 – 8.85 (m, 1H, N*H*), 8.68 (d, *J* = 8.2 Hz, 1H, N*H*), 8.52 (s, 1H, N*H*), 7.39 – 7.13 (m, 10H, Ar*H*), 4.75 – 3.96 (m, 6H, C*H*), 3.19 – 2.79 (m, 12H, CH_2).

^{13}C-NMR: (151 MHz, DMSO-d_6) δ = 171.7, 171.6, 170.5, 170.2, 166.8, 166.7, 137.2, 137.2, 129.3, 129.2, 129.2, 128.3, 128.2, 128.2, 128.1, 126.5, 126.4, 125.5, 61.2, 54.2, 54.1, 52.2, 50.9, 50.7, 38.4, 38.1, 37.7, 37.4, 22.7, 14.0, 14.0.

1.13 Cys-Phe-Cys (CFC)

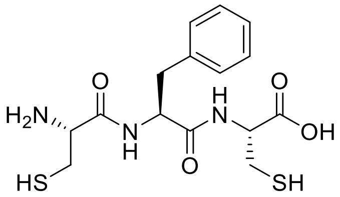

Cys-Phe-Cys was synthesized according to the general procedure for manual SPPS and purified by preparative HPLC.

Yield: 326 mg, 0.88 mmol, 37%.

HR-MS: $[M+Na]^{+}$: m/z = 394.0858 (calc. 394.0866).

^{1}H-NMR: (300 MHz, DMSO-d_6) δ = 8.70 (d, *J* = 7.9 Hz, 1H, N*H*), 8.60 (d, *J* = 7.9 Hz, 1H, N*H*), 8.53 – 7.64 (bs, 3H, NH_3^+), 7.36 – 7.18 (m, 5H, C*H*), 4.72 – 4.61 (m, 1H, C*H*), 4.43 (td, *J* = 7.4, 4.5 Hz, 1H, C*H*), 4.02 – 3.92 (m, 1H, C*H*), 3.15 – 2.72 (m, 6H, CH_2).

1.14 Cys-Gln-Cys (CEC)

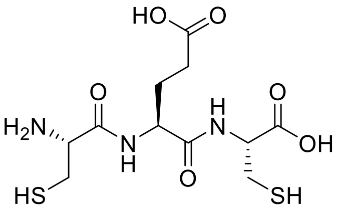

Cys-Glu-Cys was synthesized according to the general procedure for manual SPPS and purified by preparative HPLC.

Yield: 326 mg, 0.88 mmol, 37%.

HR-MS: $[M+Na]^+$: *m*/z = 354.0788 (calc. 354.0788).

^{1}H-NMR: (300 MHz, DMSO-d_6) δ = 8.68 (d, *J* = 7.5 Hz, 1H, N*H*), 8.40 (d, *J* = 7.8 Hz, 1H, N*H*), 4.47 – 4.30 (m, 2H, C*H*), 4.02 (t, J = 5.5 Hz, 1H, C*H*), 3.05 – 2.70 (m, 4H, CH_2), 2.40 – 2.26 (m, 2H, CH_2), 2.04 – 1.92 (m, 1H, CH_2-b), 1.88 – 1.76 (m, 1H; CH_2-b).

1.15 Cys-Ala-Cys (CAC)

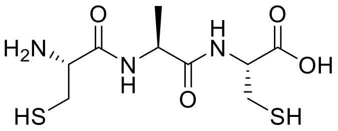

Cys-Phe-Cys was synthesized according to the general procedure for manual SPPS and purified by preparative HPLC.

Yield: 473 mg, 1.60 mmol, 66%.

HR-MS: $[M+Na]^+$: *m*/z = 296.0736 (calc. 296.0733).

^{1}H-NMR: (300 MHz, DMSO-d_6) δ = 8.71 (d, *J* = 6.9 Hz, 1H, N*H*), 8.35 (d, *J* = 7.9 Hz, 1H, N*H*), 8.23 (s, 3H,NH_3^+), 4.47 – 4.34 (m, 2H, C*H*), 4.01 (t, *J* = 5.0 Hz, 2H, C*H*), 2.93 – 2.71 (m, 4H, CH_2), 1.28 (d, *J* = 7.1 Hz, 3H, CH_3).

1.16 Cys-Ser-Cys (CsC)

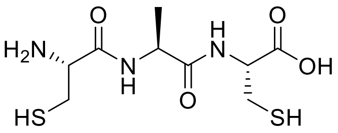

Cys-Ser-Cys was synthesized according to the general procedure for manual SPPS and purified by preparative HPLC.

Yield: 473 mg, 1.60 mmol, 66%.

HR-MS: $[M+Na]^+$: *m*/z = 334.0504 (calc. 334.0502).

^{1}H-NMR: (300 MHz, DMSO-d_6) δ = 8.70 (d, J = 7.5 Hz, 1H, NH), 8.29 (d, J = 7.8 Hz, 1H, NH), 4.48 -4.36 (m, 2H, CH), 4.05 (t, J = 5.4 Hz, 1H, CH), 3.68 – 3.58 (m, 2H, CH), 3.00 – 2.77 (m, 4H, CH_2).

2 Additional data

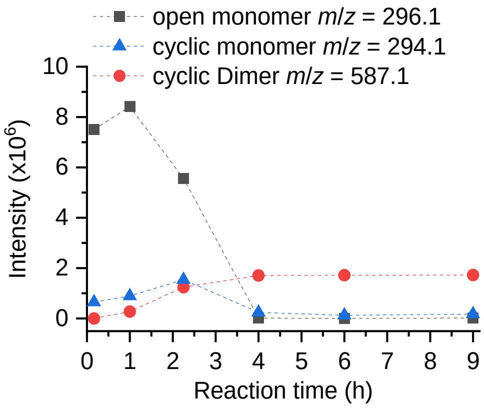

Figure S1: Time depended development of the oxidation of **CAC**. Reaction conditions were the same as for libraries in the main document. Analysis was done on an Agilent 1260 Infinity II series HPLC, which consisted of a G7129C vial sampler, a G7112B binary pump, a G7116A column oven and a G7114A variable wavelength detector. The setup was coupled to a G6125B single quadrupole mass analyzer (Agilent) and controlled by OpenLab CDS ChemStation Edition Rev. C.01.08 (Agilent). Other parameters for HPLC–MS analysis were kept the same as in the main document.

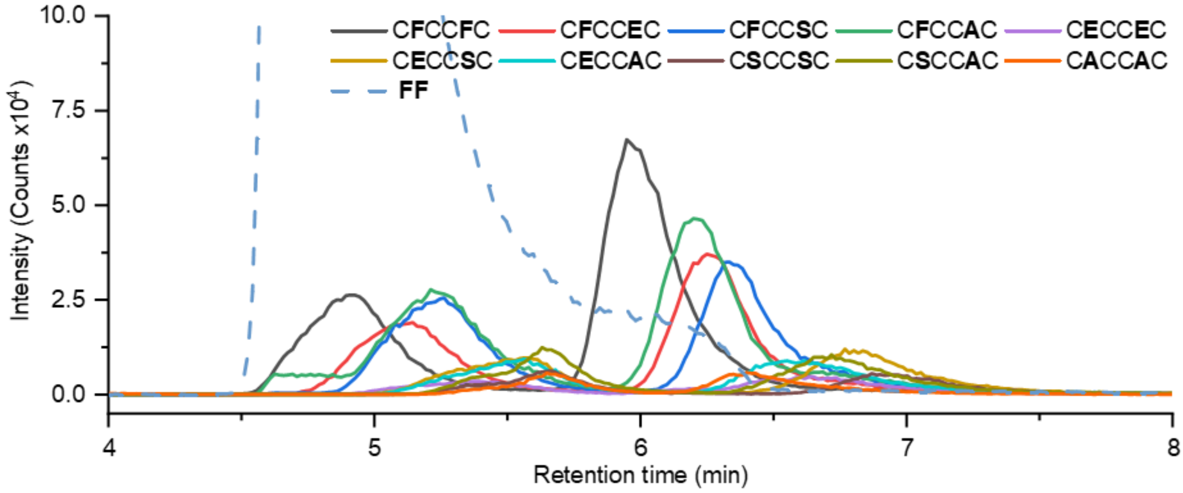

Figure S2: HPLC-MS chromatograms of library containing **FF** ([M+H]+ ions).

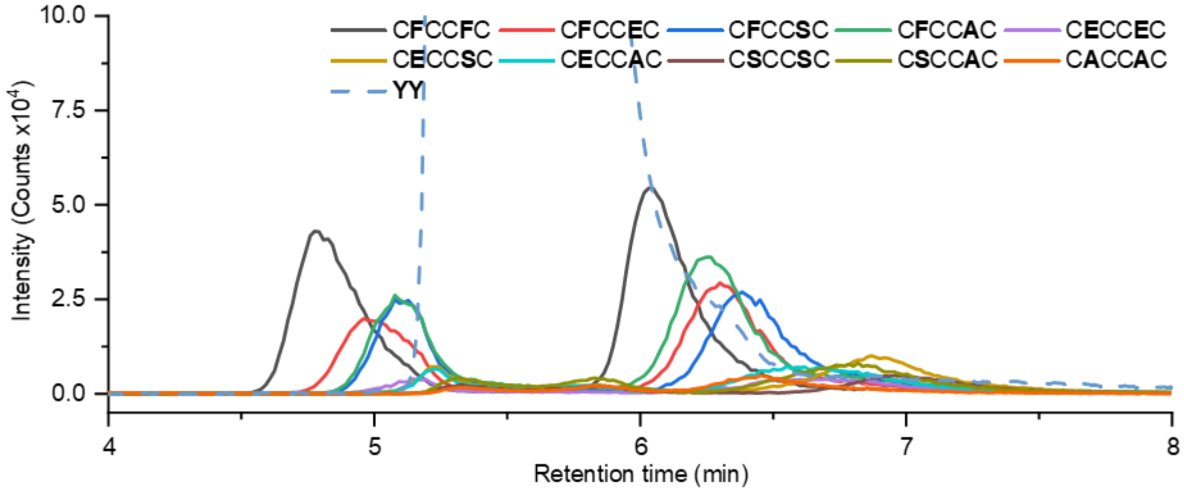

Figure S3: HPLC–MS chromatograms of library containing **YY** ($[M + H]^+$ ions).

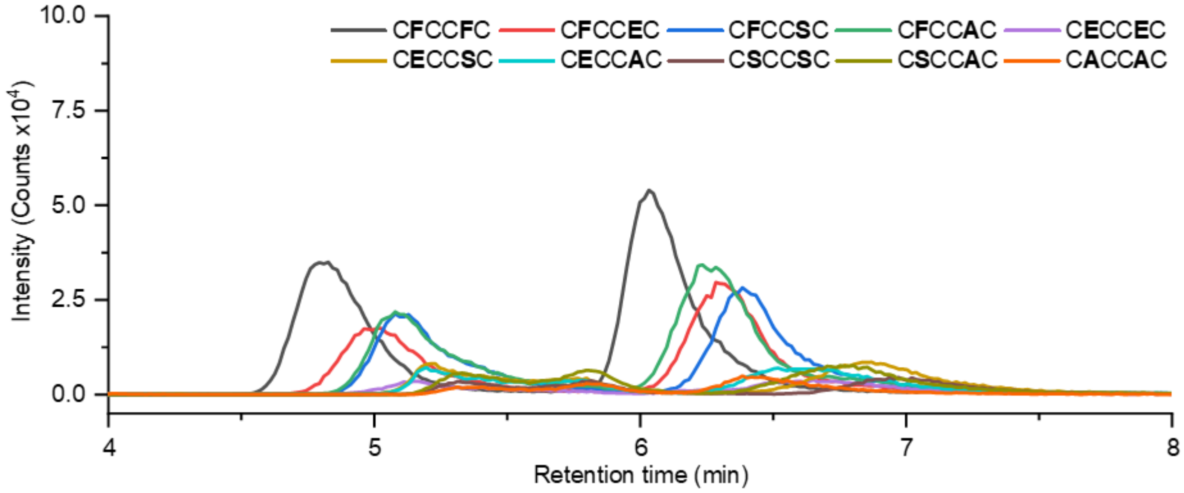

Figure S4: HPLC–MS chromatograms of library containing **AA** ($[M + H]^+$ ions).

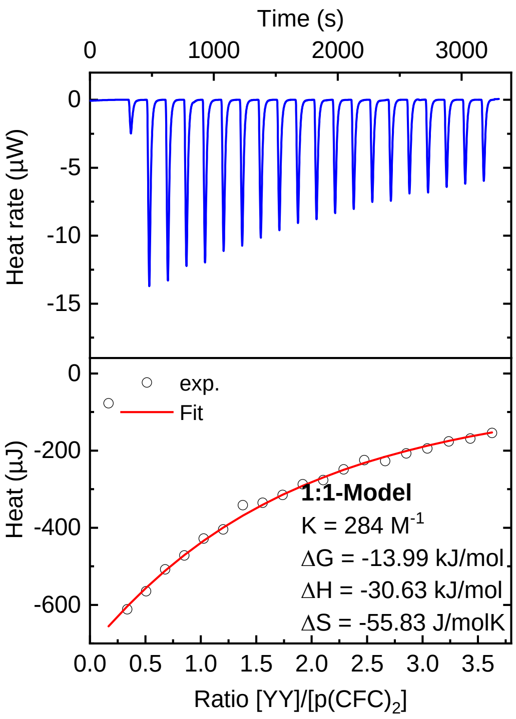

Figure S5: ITC of **YY** (30 mM) to **p(CFC)₂** (1.5 mM).

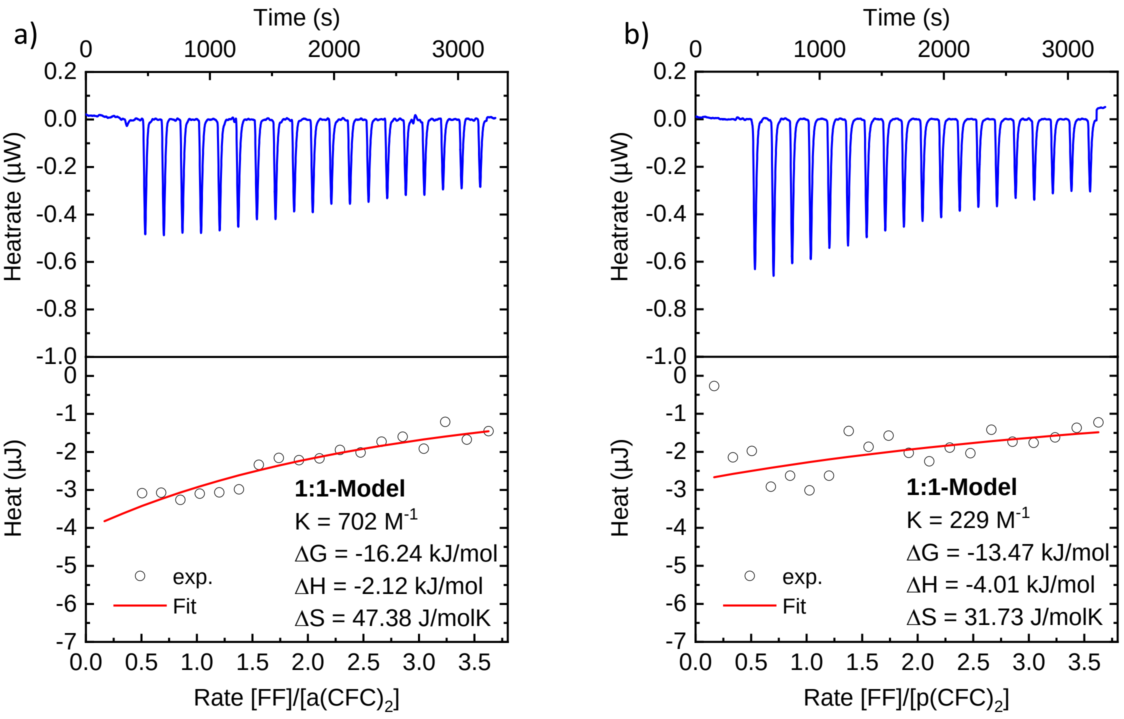

Figure S6: ITC of **FF** (5 mM) to a) **a(CFC)₂** (250 µM) and b) **p(CFC)₂** (250 µM).

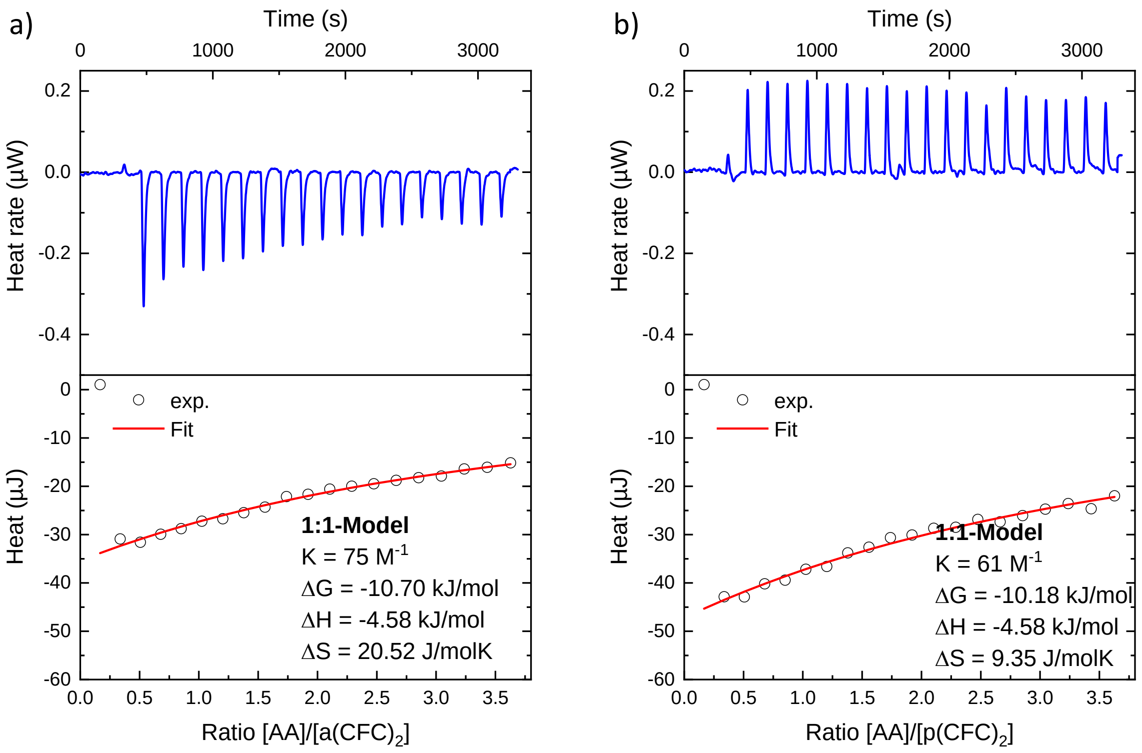

Figure S7: ITC of **AF** (30 mM) to a) **a(CFC)₂** (1.5 mM) and b) **p(CFC)₂** (250 mM).

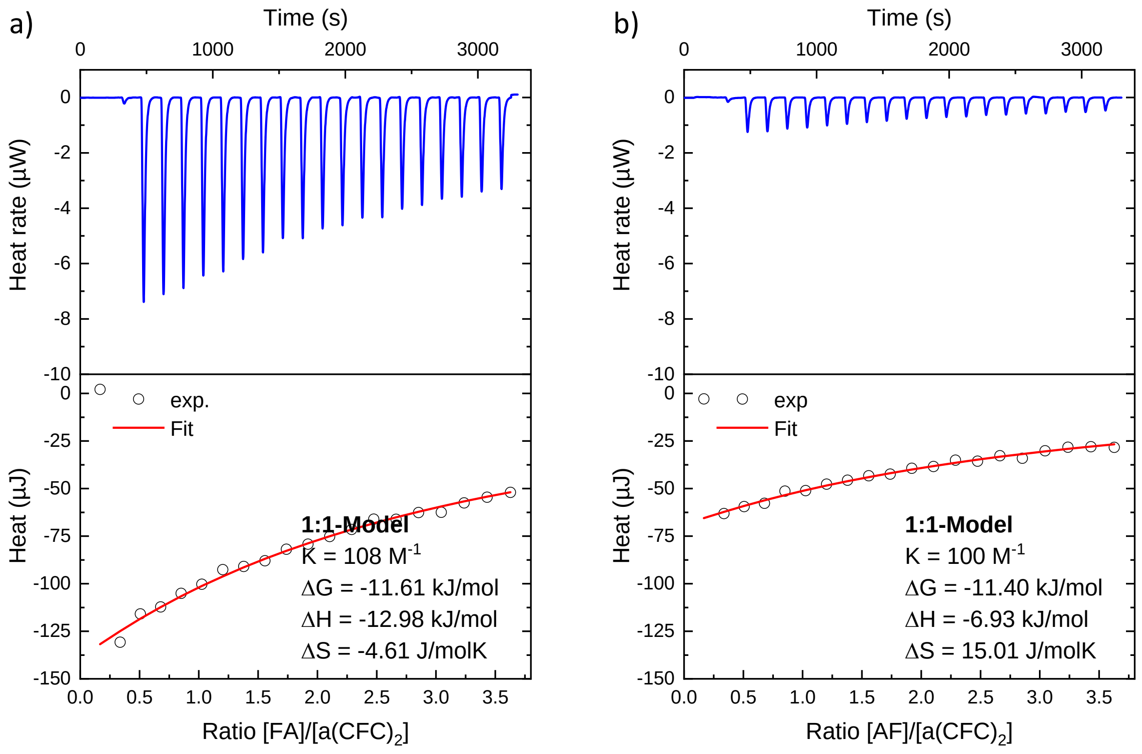

Figure S8: ITC of **FA** (30 mM) to a) **a(CFC)₂** (1.5 mM) and b) **p(CFC)₂** (1.5 mM).

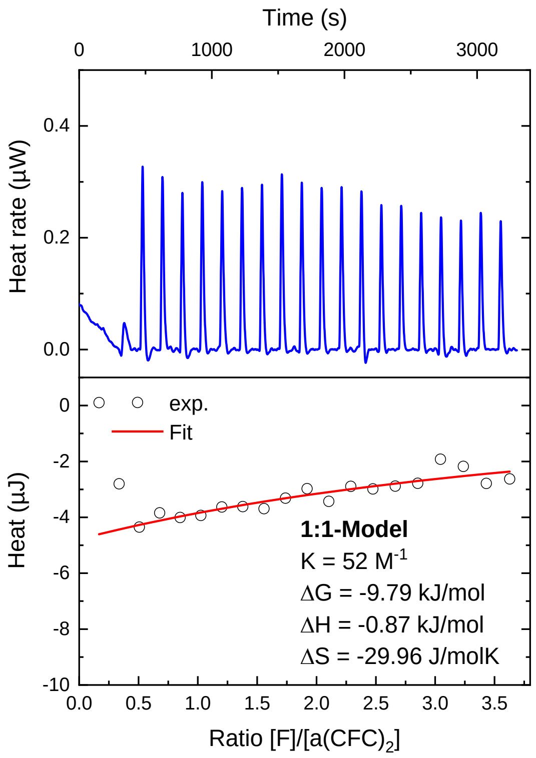

Figure S9: ITC of **F** (30 mM) to **a(CFC)$_2$** (1.5 mM).

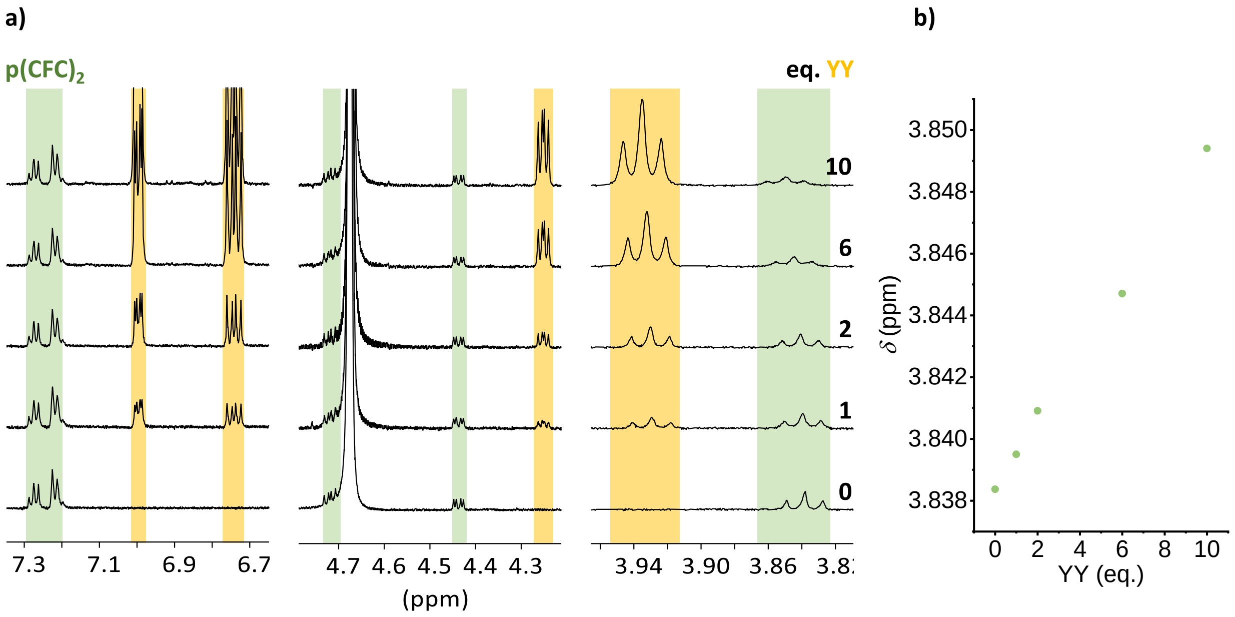

Figure S10: NMR Titration of **YY** to **p(CFC)$_2$**. a) Stacked ^{1}H NMR spectra. b) Shift of an α-proton relative to equiv of **YY**.

3 NMR-Spectra

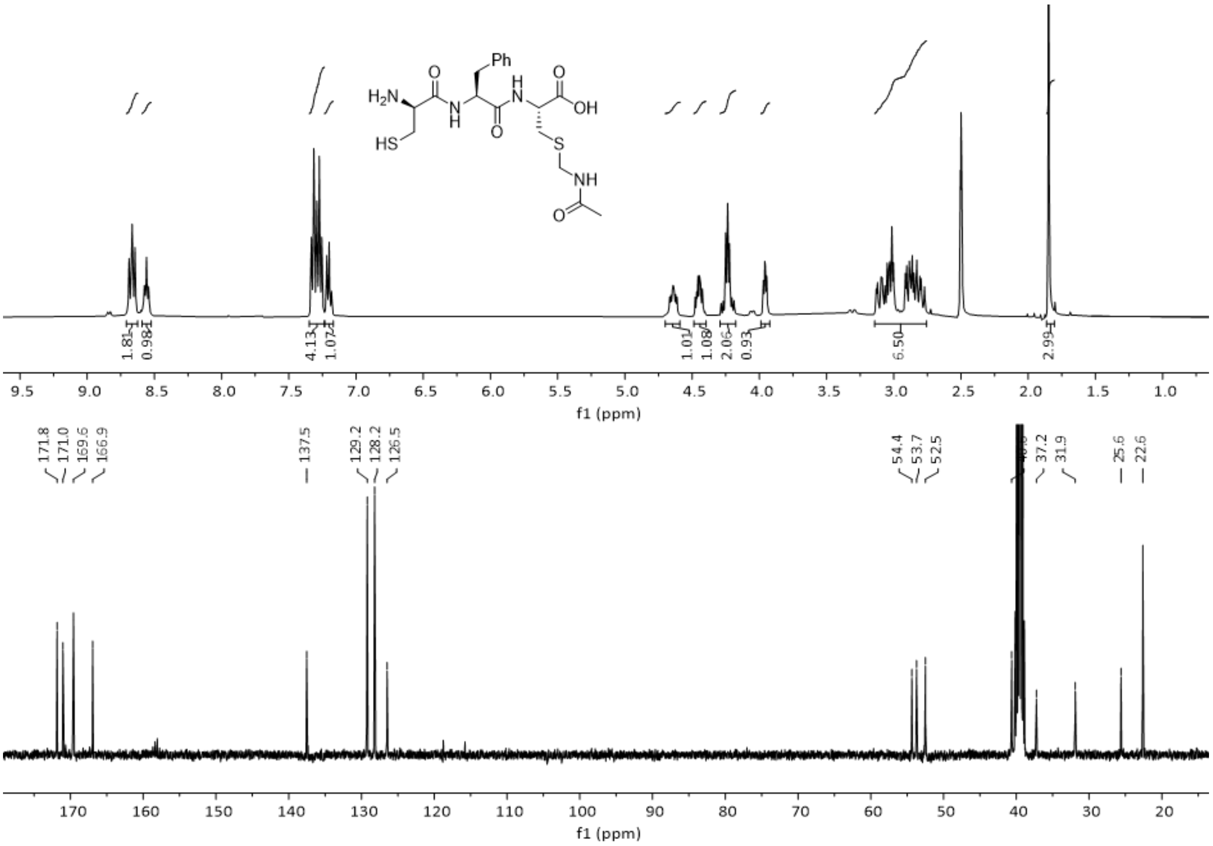

Figure S11: Top: ^{1}H NMR, bottom: ^{13}C NMR spectrum of **Cys-Phe-Cys(Acm) – CFC(Acm)**.

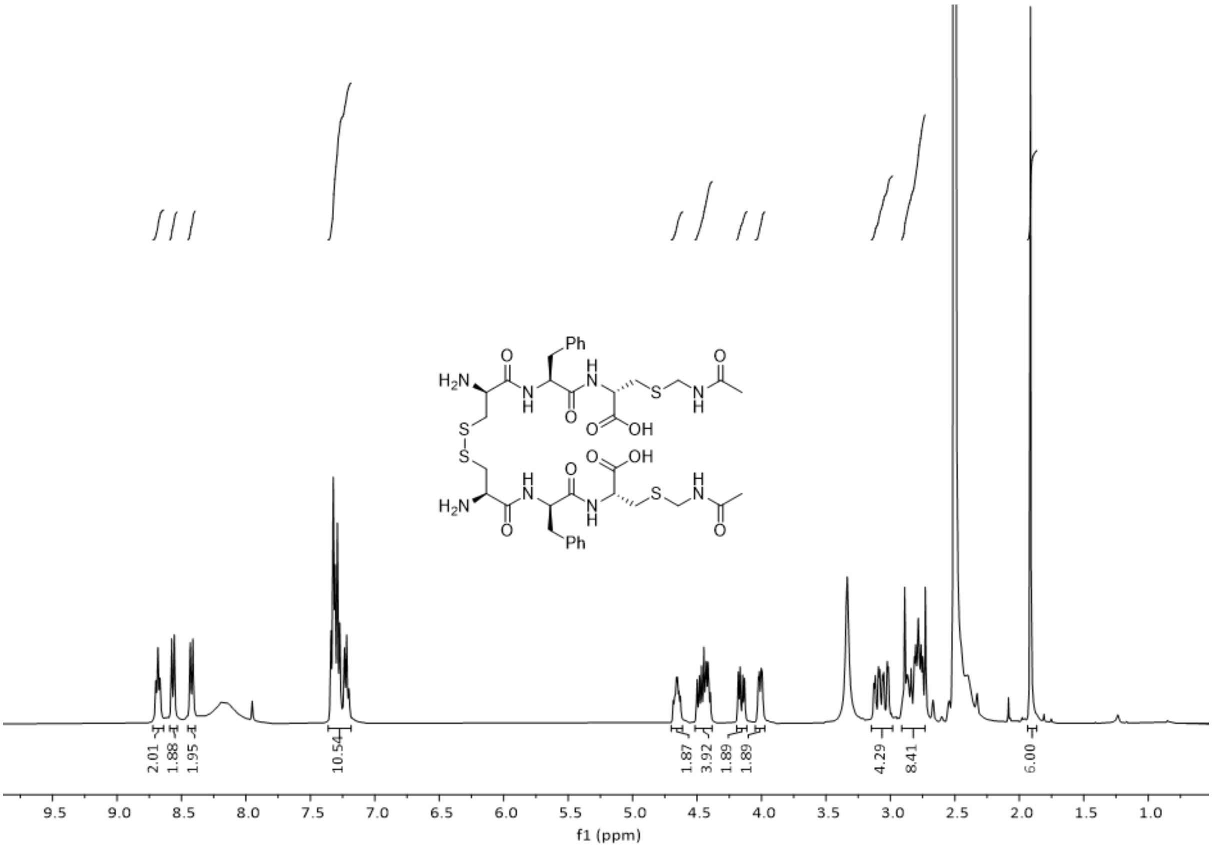

Figure S12: ^{1}H NMR spektrum von **Cys(Acm)-Phe-Cys-S-S-Cys-Phe-Cys(Acm)**.

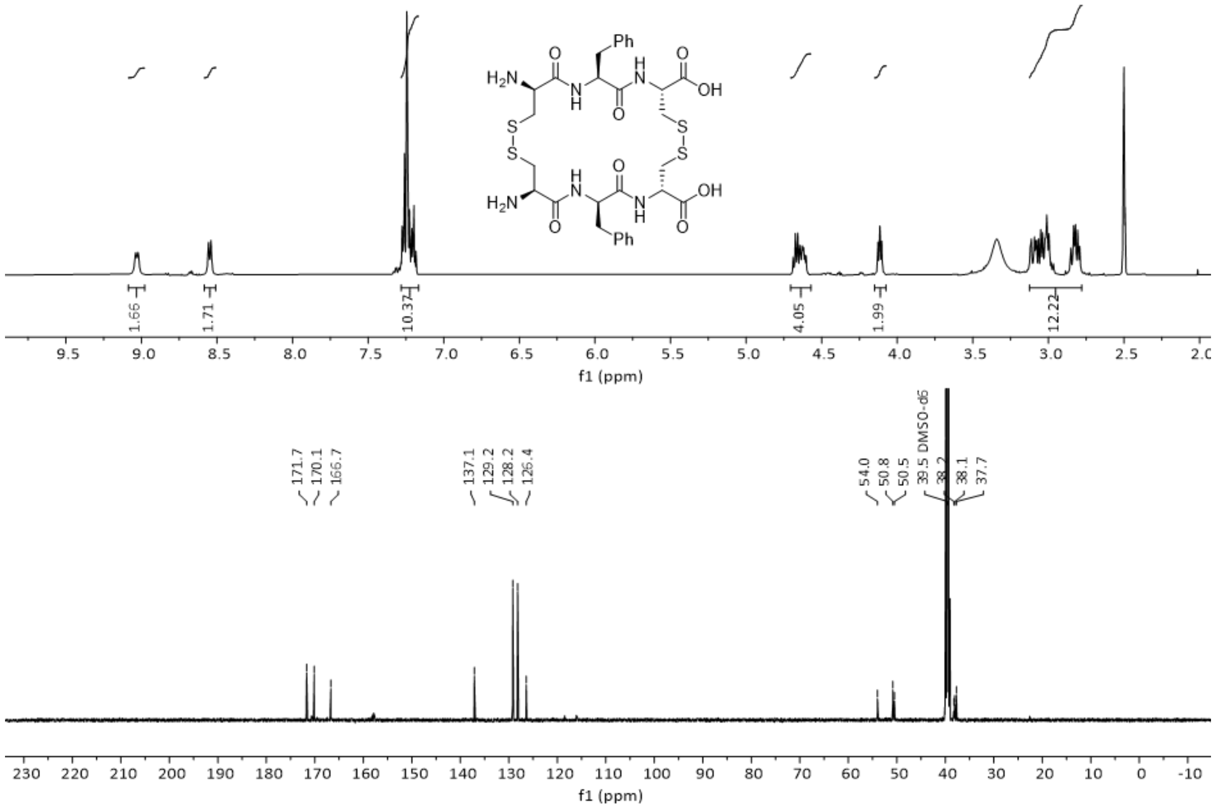

Figure S13: Top: ^{1}H NMR, bottom: ^{13}C NMR spectrum of parallel **(Cys-Phe-Cys)₂** – **p(CFC)₂**.

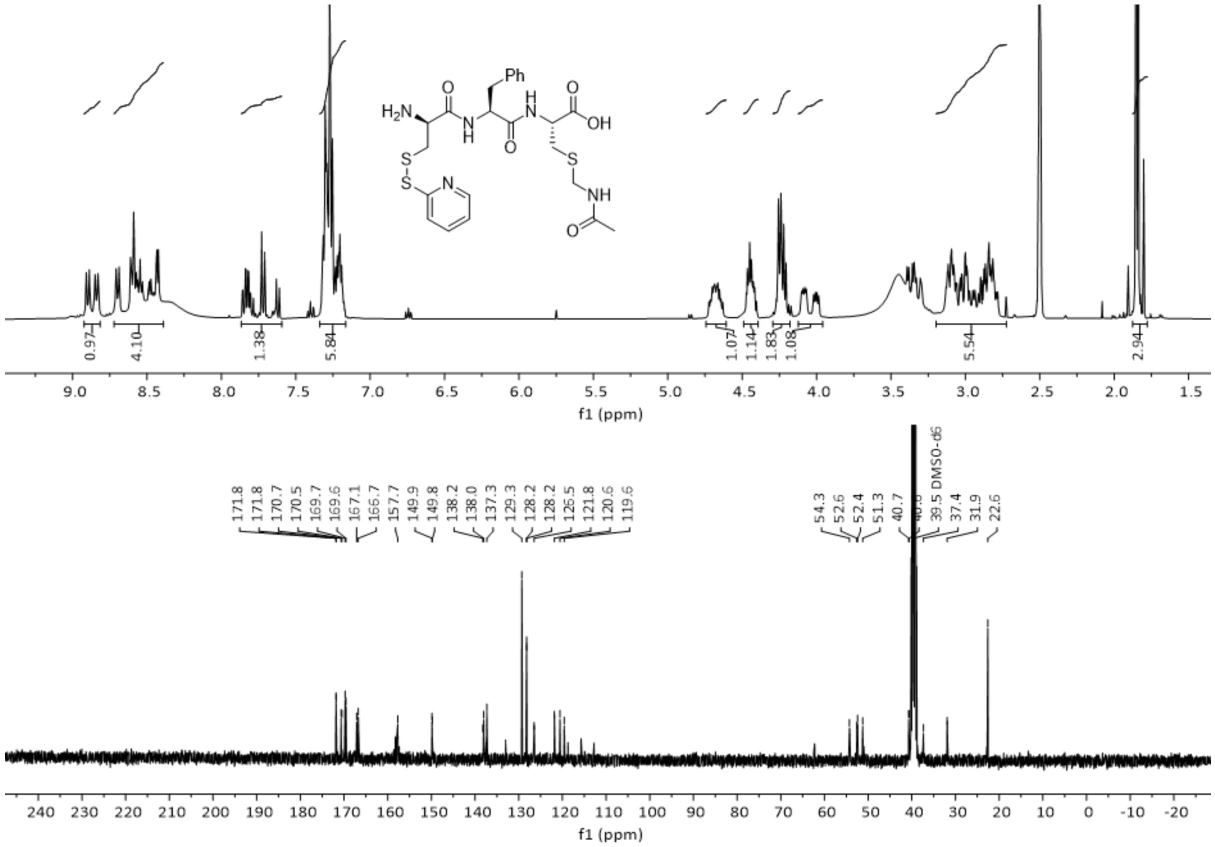

Figure S14: Top: ^{1}H NMR, bottom: ^{13}C NMR spectrum of **Cys(pyridin-2-ylthio)-Phe-Cys(Acm)**.

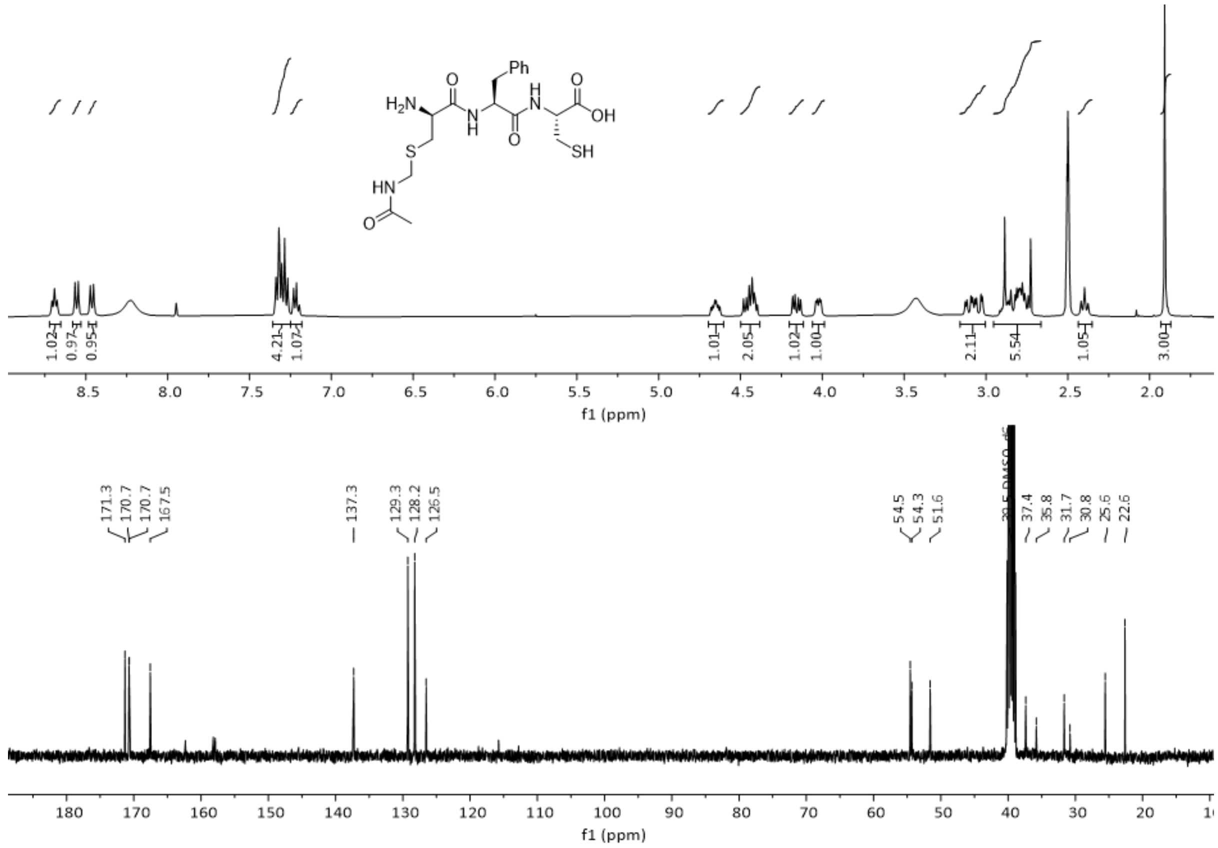

Figure S15: Top: ^{1}H NMR, bottom: ^{13}C NMR spectrum of **Cys(Acm)-Phe-Cys** – **C(Acm)FC**.

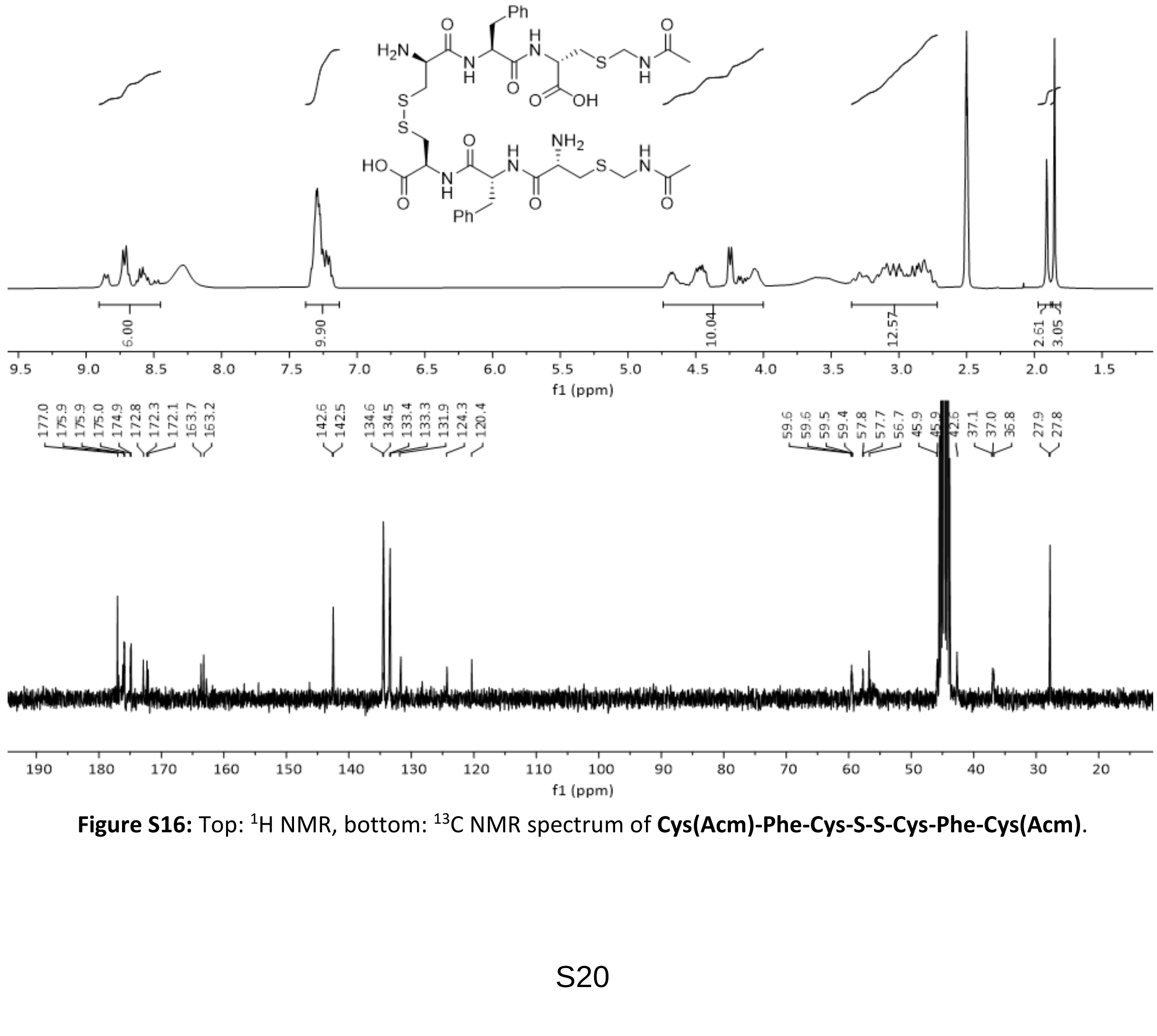

Figure S16: Top: ^{1}H NMR, bottom: ^{13}C NMR spectrum of **Cys(Acm)-Phe-Cys-S-S-Cys-Phe-Cys(Acm)**.

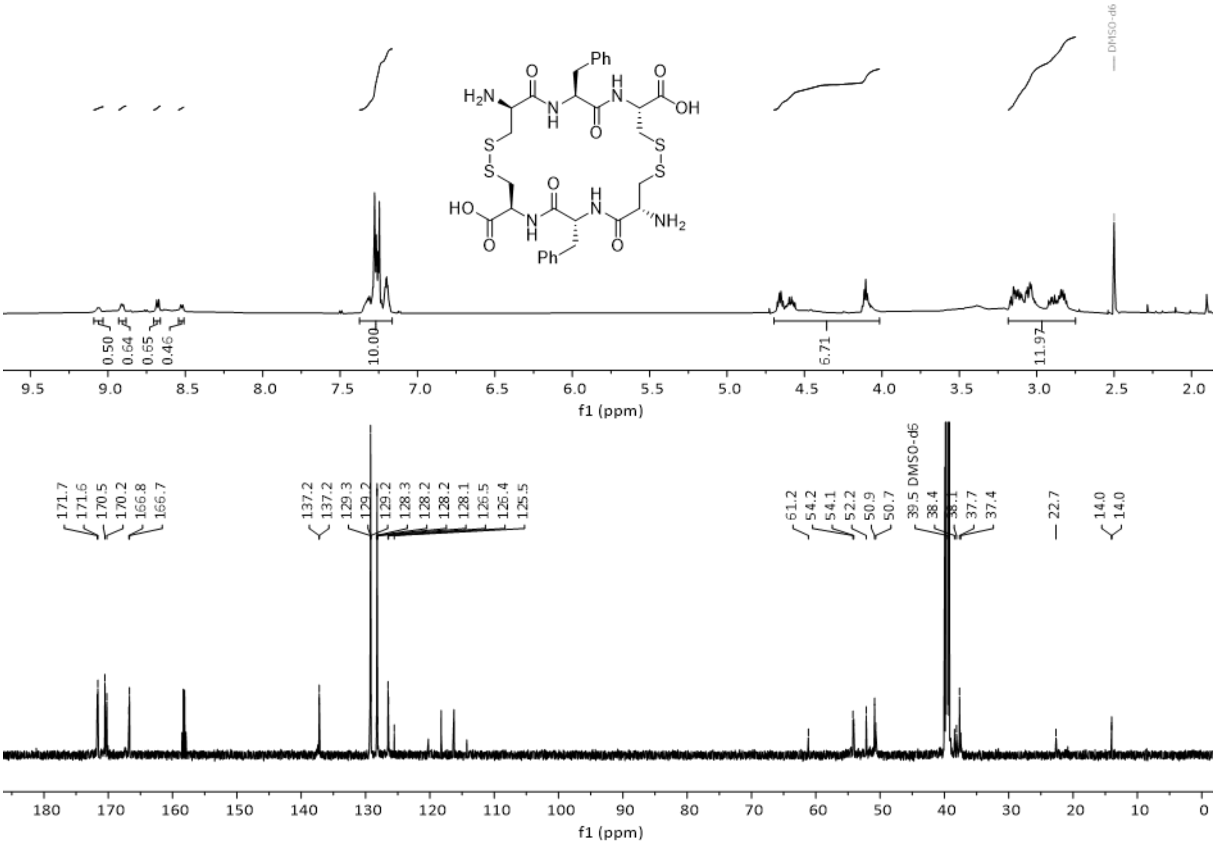

Figure SI17: Top: ^{1}H NMR, bottom: ^{13}C NMR spectrum of antiparallel **(Cys-Phe-Cys)$_2$ – a(CFC)$_2$**.

5 References

(1) Chan, W.; White, P. *Fmoc Solid Phase Peptide Synthesis: A Practical Approach*; OUP Oxford, 2000

(2) C. Lange, S.; Unsleber, J.; Drücker, P.; Galla, H.-J.; P. Waller, M.; Jan Ravoo, B. *Organic & Biomolecular Chemistry* **2015**, *13* (2), 561–569. doi:10.1039/C4OB02069C

(3) Samuel, C. S.; Lin, F.; Hossain, M. A.; Zhao, C.; Ferraro, T.; Bathgate, R. A. D.; Tregear, G. W.; Wade, J. D. *Biochemistry* **2007**, *46* (18), 5374–5381. doi:10.1021/bi700238h
